# Administration of anticoagulation strategies for portal vein thrombosis in cirrhosis: network meta-analysis

**DOI:** 10.3389/fphar.2024.1462338

**Published:** 2025-01-06

**Authors:** Hui-Jun Li, Fu-Qiang Yin, Yu-Tong Ma, Teng-Yu Gao, Yu-Ting Tao, Xin Liu, Xian-Feng Shen, Chao Zhang

**Affiliations:** ^1^ Center for Evidence-Based Medicine and Clinical Research, Taihe Hospital, Hubei University of Medicine, Shiyan, Hubei, China; ^2^ Hepatobiliary and Pancreatic Research Center, Taihe Hospital, Hubei University of Medicine, Shiyan, Hubei, China

**Keywords:** portal vein thrombosis, cirrhosis, direct oral anticoagulants, transjugular intrahepatic portosystemic shunt, low molecular weight heparin, warfarin

## Abstract

**Objectives:**

Evidences for anticoagulation strategies in cirrhotic with portal vein thrombosis (PVT) are still insufficient. This study aims to comprehensively compare the therapeutic effects of different therapeutic therapeutic measures in individuals suffering from cirrhosis with PVT, with the ultimate goal of providing evidence-based recommendations for thrombolytic therapy in this population.

**Methods:**

Starting from 20 October 2023, a comprehensive search about therapeutic strategies for portal vein thrombosis in cirrhosis was conducted on PubMed, EMBASE, and Cochrane Library.

**Results:**

19 studies were eventually incorporated into this study. Comparison with control in network meta-analysis, direct oral anticoagulants (DOACs) (RR = 2.15, 95%CI: 1.33, 3.48), LMWH (RR = 1.41, 95%CI: 1.01, 1.99), TIPS (RR = 5.68, 95%CI: 2.63, 12.24), warfarin (RR = 2.16, 95%CI: 1.46, 3.21), EBL *plus* propranolol (RR = 2.80, 95%CI: 1.18, 6.60), LMWH-DOACs sequential (RR = 7.92, 95%CI: 2.85, 21.99) and LMWH-warfarin sequential (RR = 2.26, 95%CI: 1.16, 4.42) significantly improved the incidence of complete recanalization. The anticoagulation drugs were ranked based on their SUCRA values, with the LMWH-DOACs sequential (92.7%), TIPS *plus* warfarin (91.3%), and TIPS (80.3%) emerging as the top three effective treatments.

**Conclusion:**

In this study, active anticoagulants were recommended for cirrhosis with PVT. The TIPS *plus* warfarin, LMWH-DOACs sequential, and TIPS improved the complete recanalization rate most effectively, and the EBL *plus* propranolol, heparin *plus* DOACs *plus* warfarin, and DOACs were highly recommended for increasing the incidence of partial recanalization. Warfarin and TIPS were recommended for reducing the frequency of bleeding events, while LMWH *plus* warfarin and DOACs proved to be most effective in decreasing the rate of major bleeding events. Warfarin, heparin *plus* DOACs *plus* warfarin, and DOACs demonstrated the most significant reduction in mortality rates, highlighting its potential as an effective intervention. TIPS *plus* warfarin, LMWH-DOACs sequential, and TIPS were recommended for reducing the occurrence of PVT expansion. Heparin *plus* DOACs *plus* warfarin was recommended for reducing the occurrence of hepatic encephalopathy, and protocols that involve TIPS were generally associated with a higher risk of hepatic encephalopathy. However, a longer follow-up period is necessary to comprehensively evaluate the efficacy of active anticoagulants therapy in patients with PVT in cirrhosis.

## 1 Introduction

Due to the rebalancing of hemostatic factors and alterations in portal vein status, patients with cirrhosis were predisposed to developing thrombosis in the main trunk or branches of the portal vein, which may also extend to other intra-abdominal veins (Senzolo et al., 2021). In addition, there existed a close association between portal vein thrombosis and cirrhosis, with a positive correlation observed between the severity of cirrhosis and the occurrence of this complication (Mantaka et al., 2023). Study (Pan et al., 2022) had demonstrated that the prevalence of portal vein thrombosis (PVT) in patients with liver cirrhosis was 11.18%–16.91%. Although there was no significant association between PVT and the progression of liver cirrhosis, its occurrence elevated the risk of portal hypertension and variceal bleeding, diminished hepatic perfusion, and consequently contributed to an unfavorable prognosis (Scheiner et al., 2018). In contrast, the presence of PVT can significantly contribute to increased surgical complexity and higher rates of liver transplant failure in patients with cirrhosis (Mohan et al., 2020). Therefore, addressing PVT was imperative for improving patient survival rates and reducing mortality associated with liver transplantation.

The use of conventional anticoagulation therapy, which was commonly employed in other patient populations, was currently being explored for the treatment of cirrhotic patients with PVT (Loffredo et al., 2017). Traditional anticoagulants included low molecular weight heparin (LMWH) and warfarin (Wang et al., 2021). The utilization of conventional medications, however, may give rise to significant complications associated with hemorrhaging. Consequently, direct oral anticoagulants (DOACs), such as dabigatran and factor Xa inhibitors like apixaban, edoxaban, and rivaroxaban had been increasingly employed in clinical anticoagulation therapy due to their direct inhibition of activated coagulation factors (Noronha Ferreira et al., 2019a). For patients with impaired endogenous blood agglutination ability, caution may be warranted when considering drug anticoagulation (La Mura et al., 2018). Consequently, in select cases of portal hypertension, an early transjugular intrahepatic portosystemic shunt (TIPS) procedure was performed to not only facilitate recanalization of PVT, but also ameliorate portal hypertension, thereby mitigating the risk of variceal bleeding and ascites formation (Mukund et al., 2023). The efficacy of anticoagulant therapy had been demonstrated in patients with PVT, with complete recanalization observed in 44% of cases and partial recanalization achieved in over half of the patients. Moreover, a significant improvement in the extent of PVT was noted in the majority of individuals. Additionally, several studies had indicated that therapeutic strategies not only enhances prognosis following complete recanalization but may also confer additional benefits such as reduced hepatic decompensation and mortality (Serper et al., 2021; Violi et al., 2020). Recently, Li et al. (2023) demonstrated that DOACs exhibit a reduced risk of bleeding in patients with cirrhosis. Furthermore, DOACs did not show an elevated risk of bleeding in cirrhotic patients with PVT when compared to the absence of anticoagulant therapy. However, the study did not provide an analysis based on differences in effectiveness of specific drugs.

To sum up, there was currently a lack of network meta-analysis investigating the efficacy and safety of anticoagulant therapy in patients with PVT in cirrhosis. Therefore, this study aims to comprehensively compare the therapeutic effects of different therapeutic therapeutic measures in individuals suffering from cirrhosis with PVT, with the ultimate goal of providing evidence-based recommendations for thrombolytic therapy in this population.

## 2 Materials and methods

The present study adhered to the guidelines of the Preferred Reporting Program for Systematic Reviews and Meta-analyses, including the extension statement for reporting of systematic reviews incorporating network meta-analyses (Hutton et al., 2015).

### 2.1 Search strategy

Starting from 20 October 2023, a comprehensive search was conducted on PubMed, EMBASE, and Cochrane Library. Additionally, a manual search of references from included studies was performed to supplement the electronic search. A diverse array of MeSH terms and Keywords, encompassing entry terms and free terms, constituted “liver cirrhosis”, “liver fibrosis”, “cirrhosis”, “portal vein thrombosis”, “venous thrombosis”, “anticoagulants”, “direct oral anticoagulants”, “novel oral anticoagulants”, “Factor Xa inhibitor”, “dabigatran”, “apixaban”, “edoxaban”, “rivaroxaban”, “transjugular intrahepatic portasystemic shunt”, “TIPS”, “endoscopic band ligation”, “low molecular weight heparin”, “heparin” and “warfarin”.

### 2.2 Inclusion and exclusion criteria

Subjects comprised adult patients diagnosed with PVT in cirrhosis. Cirrhosis diagnosis was established by the clinician based on laboratory tests and imaging findings, while PVT confirmation involved independent assessment by two radiologists using multi-detector computed tomography. The interventions comprised TIPS, endoscopic band ligation (EBL), DOACs (dabigatran, apixaban, edoxaban, or rivaroxaban), conventional medications (LMWH or warfarin), or a combination of these interventions. The Control group was control or other active anticoagulants. The primary outcomes evaluated were complete recanalization and partial recanalization. The secondary outcomes encompassed bleeding, major bleeding, mortality, PVT extension, and hepatic encephalopathy. The studies included randomized controlled trials (RCTs) and cohort studies.

The exclusion criteria were established to include individuals with a life expectancy of fewer than 6 months, patients diagnosed with malignant PVT, underlying primary hematologic disorders or Budd-Chiari syndrome, membranous obstruction of the inferior vena cava or preexisting extrahepatic thrombosis. Additionally excluded were subjects receiving unspecified medications or having incomplete data records and non-English language publications.

### 2.3 Study selection and data extraction

Two researchers independently conducted a systematic literature review, strictly adhering to the predefined inclusion criteria for data extraction. Any disagreements were resolved through comprehensive discussions involving a third researcher. The eligible studies provided the following extracted information or data: the first author’s name and publication year, country where the study was conducted, study type, number of subjects included, intervention details, demographic characteristics such as mean age and gender distribution among participants, Child-Pugh classification status, model for end-stage liver disease (MELD) score values, number of detected esophageal varices, international normalization ratio (INR) for anticoagulation therapy administration, platelet count measurements in individuals undergoing anticoagulation therapy (10^9^/L), creatinine levels expressed in µmol/L units, and duration of follow-up period measured in months.

### 2.4 Quality assessment of including studies

To evaluate trial validity, two independent reviewers utilized the risk-of-bias (ROB) 2.0 tool (Sterne et al., 2019) to assess the risk of bias in RCTs and employed the risk of bias in non-randomized studies-of interventions (ROBIN-I) tool (Sterne et al., 2016) for non-RCTs. In case of any discrepancies, a third reviewer would provide comprehensive judgment. The risk of bias in the included RCTs was assessed based on random allocation sequence, allocation concealment, blinding of patients and personnel, blinding of outcomes assessment, incomplete outcome data, selective reporting, and other potential sources of bias. The risk levels were categorized as high, medium, low or unknown. The risk of bias in the non-RCTs study was assessed across seven domains: confounding, participant selection, intervention classification, deviations from intended interventions, missing data, outcome measurement, and reported result selection. The overall risk of bias was categorized as low, moderate, serious, critical or unknown.

### 2.5 Statistical analysis

The dichotomous outcomes were reported as relative risks (RR) accompanied by 95% confidence intervals (CI). Heterogeneity among studies was evaluated using the chi-square test (P< 0.1) and the I^2^ statistic. A significant heterogeneity exceeding 40% (I^2^ ≥ 40%) indicated the adoption of a random effects model, while a fixed effects model was employed when I^2^ was less to 40%. The network meta-analysis utilized the design-treatment interaction model of processing design. The observed inconsistency between direct and indirect evidence suggested that the transferability of results was not readily apparent through node segmentation methodology. To summarize the probabilities, we used the area under the cumulative ranking curve (SUCRA) to provide a summary of the cumulative rankings. In addition, the Child-Pugh C >20% was utilized as a sensitivity analysis. STATA 15.0 and R 4.2.2 software were employed for all statistical analysis.

## 3 Results

### 3.1 Study selection

**FIGURE 1:**
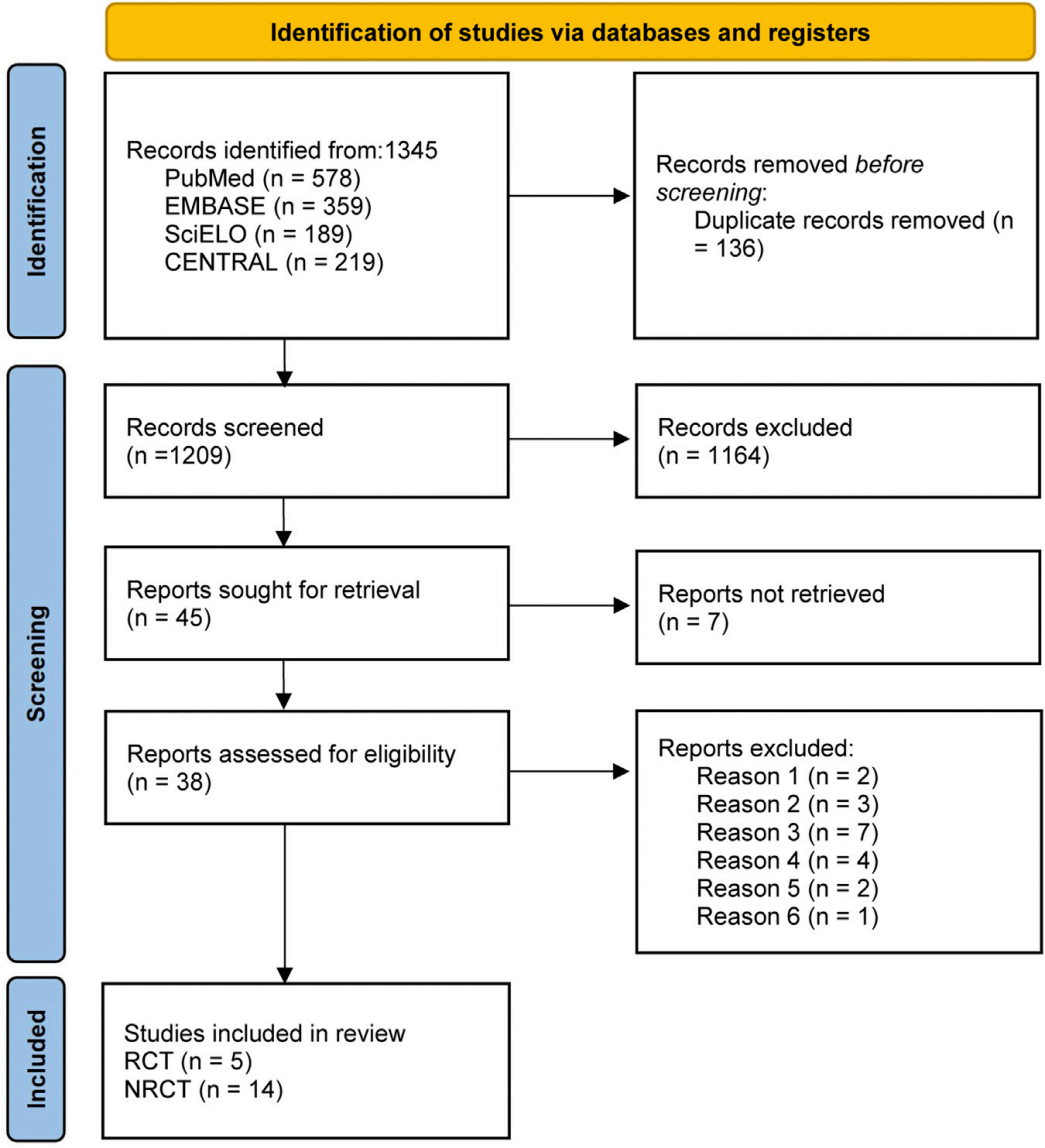
Literature screening for inclusion of studies. Note: SciELO, Scientific Electronic Library Online; CENTRAL, Cochrane Central Register of Controlled Trials; Reason 1, subjects with a life expectancy of less than 6 months; Reason 2, patients diagnosed with malignant PVT; Reason 3, underlying primary hematologic disorders or Budd-Chiari syndrome; Reason 4, membranous obstruction of the inferior vena cava or preexisting extrahepatic thrombosis; Reason 5, missing data; Reason 6, publications not written in English; RCT, randomized controlled trial; NRCT, non-randomized controlled trial.

A total of 1,345 articles were retrieved for initial screening, with the exclusion of 136 duplicate articles as an initial step. Subsequently, following a thorough evaluation of titles and abstracts, an additional 1,164 articles were excluded from consideration. A comprehensive review of the articles resulted in the exclusion of 19 articles that did not meet the inclusion criteria for this study. These exclusions included 2 articles involving subjects with a life expectancy of less than 6 months, 3 articles involving subjects diagnosed with malignant PVT, 7 articles involving subjects presenting membranous obstruction of the inferior vena cava or preexisting extrahepatic thrombosis, 4 articles lacking specification on the specific drugs used by the subjects, and 2 articles containing missing data. Additionally, no valid data could be obtained through communication with the author, and 1 non-English publication was excluded. 19 studies (Ai et al., 2020; Caracciolo et al., 2013; Chen et al., 2016; Chung et al., 2014; Florescu et al., 2021; Gao et al., 2023; Garcovich et al., 2011; Joseph and Rejeski, 2020; Luo et al., 2015; Lv et al., 2021; Lv et al., 2018; Nagaoki et al., 2018; Naymagon et al., 2021; Noronha Ferreira et al., 2019b; Pettinari et al., 2019; Senzolo et al., 2012; Wang et al., 2016; Zhang et al., 2023; Zhou et al., 2020) were eventually incorporated into this study, comprising 5 RCTs (Gao et al., 2023; Luo et al., 2015; Lv et al., 2018; Wang et al., 2016; Zhou et al., 2020) and 14 non-RCTs (Ai et al., 2020; Caracciolo et al., 2013; Chen et al., 2016; Chung et al., 2014; Florescu et al., 2021; Garcovich et al., 2011; Joseph and Rejeski, 2020; Lv et al., 2021; Nagaoki et al., 2018; Naymagon et al., 2021; Noronha Ferreira et al., 2019b; Pettinari et al., 2019; Senzolo et al., 2012; Zhang et al., 2023). The specific screening process was depicted in Figure 1.

### 3.2 Characteristics of including studies

The analysis comprised a total of 1,745 participants, with comprehensive details provided in Table 1. The study cohort was recruited from China, Italy, Korea, Japan, USA, Spain and Romania. The majority of the participants were aged over 45 years old and predominantly male. The largest number of subjects was observed in Child-Pugh class B. Four studies did not explicitly report the sample size for subjects in Child-Pugh class C, while three studies only included a limited number of control group subjects classified as Child-Pugh class C. MELD scores consistently exceeded 10 and remained above 5 for all participants. Esophageal varices were present in approximately 10% of the overall subject population. Anticoagulation therapy demonstrated an INR predominantly above 1.0, with only one study reporting a value below this threshold. The majority of subjects displayed platelet counts exceeding 95×10^9^/L and creatinine levels surpassing 70 μmol/L, with a minimum follow-up duration of 6 months.

**TABLE 1:** Summary basic characteristics of included studies.

Included studies	Year	Location	Study design	Sample size	Anticoagulant	Age (y)	Gender (M/F)	Child-Pugh (A/B/C)	MELD score	Esophageal varices	INR	Platelet countx109 /L	Creatinine (µmol/L)	Follow-up period (m)
Garcovich	2011	Italy	Retrospective cohort/Abstract	30	LMWH	NR	NR	NR	NR	NR	NR	NR	NR	6
					Control	NR	NR	NR	NR	NR	NR	NR	NR	NR
Senzolo	2012	Italy	Prospective cohort	56	Nadroparin (95 U/Kg/d)	55.5±5	25/10	11/16/8	12.6±3.7	32/3	1.4±0.6	78.3±36.5	75.4±26.2	21.6±8.5
					Control	52.3±4	13/8	5/9/7	13.7±3.6	20/1	1.4±0.23	79.6±40	82±30.3	24.5±8.2
Caracciolo	2013	Italy	Retrospective cohort/Abstract	27	LMWH	NR	NR	NR	NR	NR	NR	NR	NR	3-6
					Control	NR	NR	NR	NR	NR	NR	NR	NR	3-6
Chung	2014	Korea	Retrospective cohort	28	Warfarin 2.7 mg/d(mean)	59.4±12	10/4	6/8/0	NR	NR	1.90±0.41	NR	NR	3.8±3.1
					Control	58.7±13.2	11/3	7/6/1	NR	NR	1.43±0.18	NR	NR	3.6±3
Luo	2015	China	Randomized controlled trial	73	TIPS	50.8±13.6	19/18	0/25/12	14.2±6.5	NR	1.5±3.5	114.0±124.5	73.0±19.1	22.8±7.7
					EBL *plus* propranolol	49.5±14.0	24/12	0/24/12	15.9±5.7	NR	1.4±0.3	105.8±78.6	72.2±19.2	20.9±8.9
Chen	2016	China	Retrospective cohort	66	Warfarin	45±12.3	23/7	6/17/5	9.9±4.04	30/0	1.49±0.66	177.7±118.7	73.5±13.9	33.2±29.2
					Control	47.9±10.6	24/12	8/21/2	8.9±3.01	36/0	1.26±0.18	142.8±99.8	75.8±18.8	25.9±23
Wang	2016	China	Randomized controlled trial	64	TIPS *plus* Warfarin	54.5± 12.9	17/14	12/17/2	10.6±2.9	NR	NR	NR	NR	12
					TIPS	55.0± 12.2	21/22	12/15/6	10.9±3.1	NR	NR	NR	NR	12
Nagaoki	2018	Japan	Retrospective cohort	50	Danaparoid, 2500 U/d for 2 weeks+16 received edoxaban,; 4 received edoxaban, 60 mg, qd	69 (53-74)	7/13	15/5/0	NR	16/4	NR	117 (46-238)	NR	6
					Danaparoid, 2500 U/d for 2 weeks+ warfarin,INR goal 1.5-2.0	67 (24-83)	17/13	15/10/5	NR	26/4	NR	98 (31-416)	NR	6
Lv	2018	China	Randomized controlled trial	49	TIPS	49 (46-62)	13/11	9/13/2	12 (9-13)	22/2	1.40 (1.22-1.60)	NR	71.0 (60.5-76.8)	30.9 (21.6-42.5)
					EBL *plus* propranolol	46 (38-56)	16/9	10/14/1	10 (9-12)	21/4	1.33 (1.16-1.54)	NR	79.0 (66.0-95.5)	30.4 (24.6-39.0)
Noronha	2019	Portugal	Prospective cohort	80	15 received LMWH; 22 received Warfarin, INR goal 2-3	59±8	28/9	12/16/9	14±6	33/4	1.4±0.3	111.0±65.1	1.0±0.5 mg/dl	25.5
					Control	60±10	25/18	9/18/16	16±7	36/7	1.4±0.2	96.9±24.6	1.0±0.7 mg/dl	NR
Pettinar	2019	Italy	Retrospective cohort	182	56 received LMWH; 15 received fondaparinux;10 received VKAs	57.9±11.1	56/25	43/33/5	NR	NR	1.1±0.3	84.8±57.2	NR	19(3-94)
					Control	57.7±11.3	74/27	37/45/19	NR	NR	1.5±0.5	99.3±81.8	NR	19(3-94)
Ai	2020	China	Prospective cohort	80	26 received rivaroxaban,20 mg, qd; 14 received dabigatran, 150 mg bid	56.1±16.1	26/14	7.2±1.52	NR	NR	0.9±1.1	96.9±24.6	NR	6
					Control	52.3±19.4	24/16	7.4±1.67	NR	NR	0.9±0.8	102±20.7	NR	6
Naymagon	2020	USA	Retrospective cohort	214	Warfarin, INR goal 2-3;enoxaparin, 1 mg/kg,bid; rivaroxaban, 20 mg/d; apixaban, 5 mg, bid;dabigatran, 150 mg, bid	60 (54-67)	52/34	21/42/23	10 (7-13)	NR	1.3 (1.2-1.4)	90 (69-134)	0.9 (0.7-1.2)	21 (11-44)
					Control	60 (54-66)	90/38	31/57/40	11 (7-15)	NR	1.3 (1.2-1.4)	82 (57-130)	0.9 (0.8-1.2)	29 (14-53)
Joseph	2020	USA	Retrospective cohort/Abstract	16	DOACs	61 (59-61)	3/2	3/2/0	8 (7-11)	NR	NR	NR	NR	12
					Warfarin	55 (54-60)	10/1	2/8/1	13 (10-18)	NR	NR	NR	NR	12
Zhou	2020	China	Randomized controlled trial	64	Nadroparin, 1month *plus* warfarin, INR goal 2-3	55±9	21/11	6.51±1.27	9.13 ±3.39	26/6	1.3±0.2	126.2±170.9	60.9±16.0	6
					Control	53±10	21/11	6.81±1.44	10.00±3.65	27/5	1.4±0.2	134.6±137.5	65.8±16.1	6
Lv	2021	China	Prospective cohort	396	Heparin 8000-12,000 U/d 5 days *plus* warfarin INR goal 2-3; heparin 8000-12,000 U/d 5 days *plus* warfarin or enoxaparin 4000-8000 IU/d or Rivaroxaban 10 mg/d	47.2±11.3	36/27	33/27/3	10.3±2.9	NR	1.29±0.29	221.1 ±167.1	0.89 ±0.18 mg/dl	43.1 (22.9-59.2)
					Control	53.9 ±12.2	31/17	13/25/10	12.6±3.8	NR	1.46±0.31	141.6±117.8	0.96±0.26 mg/dl	29.8 (13.5-50.4)
					TIPS	53.6±11.9	51/37	22/51/15	12.5±3.5	NR	1.46±0.30	90.7 ±93.6	0.99±0.23 mg/dl	21.4 (15.6-26.9)
					TIPS *plus* Warfarin	52.3±11.1	120/77	67/113/17	11.5±2.9	NR	1.39±0.30	140.1±126.6	0.98± 0.24 mg/dl	41.4 (24.4-52.9)
Florescu	2021	Romania	Retrospective cohort	107	Enoxaparin 200 U/kg *plus* Enoxaparin or VKA INR goal 2-2.5	53(23-73)	29/25	13/40/1	NR	51/3	NR	NR	NR	32 (3-109)
					Control	55.65 (25-75)	25/28	14/37/2	NR	47/6	NR	NR	NR	32 (3-109)
Zhang	2023	China	Retrospective cohort	77	Warfarin INR goal 1.5-2.5 (n=6), nadroparin 4100 U qd (n=2), heparin 12,500 U qd (n=1), rivaroxaban 20 mg qd (n=3), rivaroxaban 10 mg qd (n=14), edoxaban 30 mg qd (n=1)	60.4±12.3	18/9	15/11/1	5.2±4.0	NR	1.2±0.1	97.0 (69.0-195.0)	71.6 (57.8-93.6)	10
					Control	59.0±13.0	26/24	16/24/7	6.4±5.5	NR	1.3±0.2	74.0 (52.0-102.0)	69.0 (60.0-93.8)	10.5
Gao	2023	China	Randomized controlled trial	86	Nadroparin calcium-warfarin sequential	56.54±9.62	23/20	15/26/2	9.49±1.80	42/1	1.26±0.17	116.58±75.46	62.09±11.65	6
					Control	55.58±10.62	27/16	15/27/1	10.23±2.23	43/0	1.31±0.20	108.93±89.60	62.04±14.80	6

Note: MELD, model for end-stage liver disease; VKA, vitamin K antagonists; INR, international normalization ratio; TIPS, transjugular intrahepatic portal system shunt; DOACs, direct oral anticoagulants; LMWH, low molecular weight heparin; USA, The United States of America; NR, not reported.

### 3.3 Quality evaluation

Among the 19 studies (Ai et al., 2020; Caracciolo et al., 2013; Chen et al., 2016; Chung et al., 2014; Florescu et al., 2021; Gao et al., 2023; Garcovich et al., 2011; Joseph and Rejeski, 2020; Luo et al., 2015; Lv et al., 2021; Lv et al., 2018; Nagaoki et al., 2018; Naymagon et al., 2021; Noronha Ferreira et al., 2019b; Pettinari et al., 2019; Senzolo et al., 2012; Wang et al., 2016; Zhang et al., 2023; Zhou et al., 2020) enrolled in this study, a total of 5 were RCTs (Gao et al., 2023; Luo et al., 2015; Lv et al., 2018; Wang et al., 2016; Zhou et al., 2020). The quality assessment of RCTs was presented in Supplementary Table S1, while the quality assessment of non-RCT studies was found in Supplementary Table S2. The RCT included only one study (Lv et al., 2018) with an unknown risk of bias in outcome measurement, which was assessed as moderate. Among the non-RCTs, four (Florescu et al., 2021; Garcovich et al., 2011; Nagaoki et al., 2018; Pettinari et al., 2019) were deemed to have a low risk of bias due to confounding, while three (Chung et al., 2014; Lv et al., 2021; Senzolo et al., 2012) were considered to have a medium risk of bias due to deviations from intended interventions. In terms of bias due to missing data, one study (Caracciolo et al., 2013) was categorized as having a moderate risk, while another study (Chen et al., 2016) was deemed to possess a high risk. Two studies (Caracciolo et al., 2013; Garcovich et al., 2011) were identified as exhibiting a high risk of bias in selection of the reported result, whereas the remaining studies were considered to have a moderate risk. All NRCT demonstrated low risk in terms of Bias in selection of participants into the study. The three aspects related to bias in classification of interventions and Bias in measurement of outcomes were found to be at low risk.

### 3.4 Primary outcomes

#### 3.4.1 Complete recanalization

**FIGURE 2:**
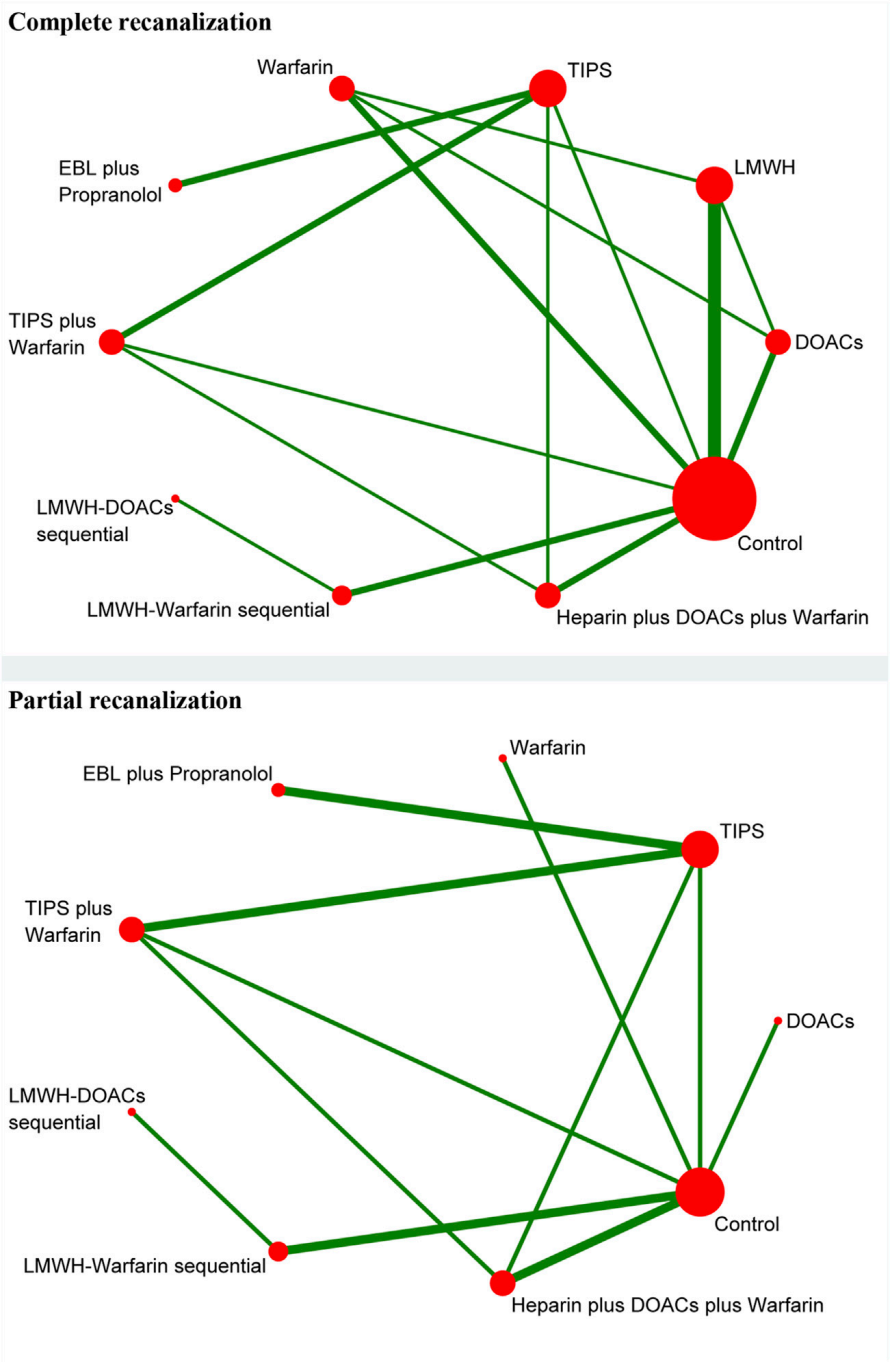
Network plot for complete and partial recanalization.

**FIGURE 3:**
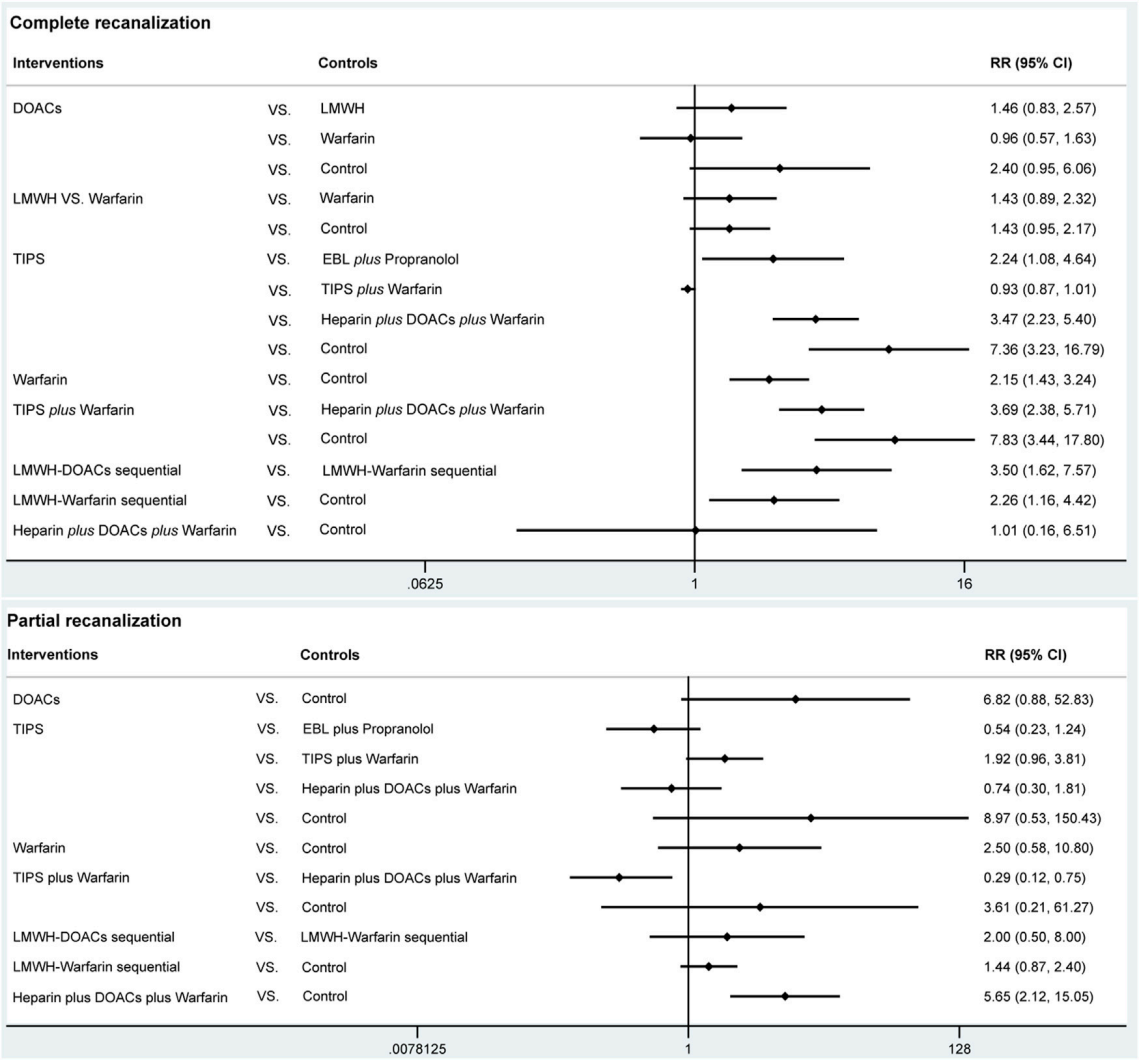
Traditional paired meta-analysis for complete and partial recanalization.

**FIGURE 4:**
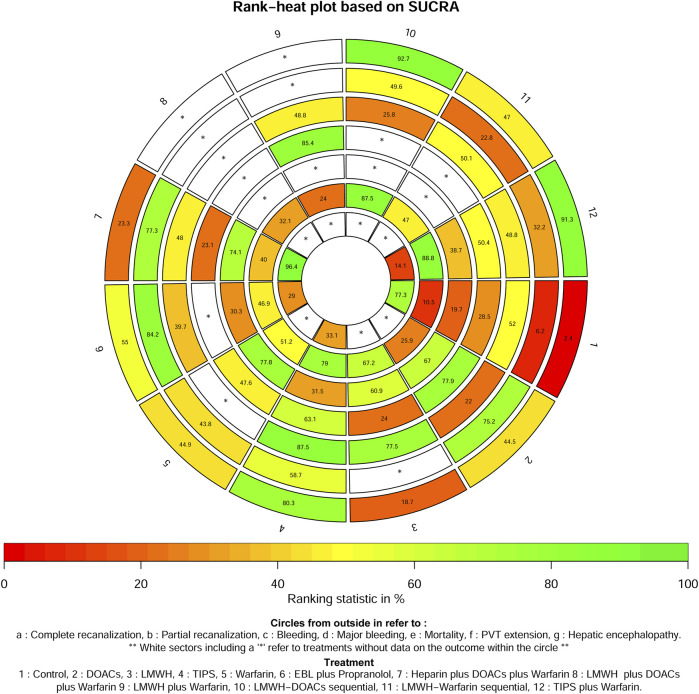
Rank−heat plot based on SUCRA for all outcomes.

A total of 14 studies (Ai et al., 2020; Caracciolo et al., 2013; Chung et al., 2014; Gao et al., 2023; Garcovich et al., 2011; Luo et al., 2015; Lv et al., 2021; Lv et al., 2018; Nagaoki et al., 2018; Naymagon et al., 2021; Senzolo et al., 2012; Wang et al., 2016; Zhang et al., 2023; Zhou et al., 2020) with 1,275 patients reported data on complete recanalization, and the correlation network diagram was illustrated in Figure 2. The traditional paired and network meta-analysis of complete recanalization was presented in Table 2 and Figure 3, showcasing the results of the study. The effect of active therapeutic strategies on the rate of complete recanalization was favorable than that of control (RR = 1.95, 95%CI: 1.37, 2.78). In direct comparisons, the TIPS group showed significantly higher rates compared to the EBL *plus* propranolol group (RR = 2.24, 95%CI: 1.08, 4.64), the heparin *plus* DOACs *plus* warfarin group (RR = 3.47, 95%CI: 2.23, 5.40), and the control group (RR = 7.36, 95%CI: 3.23,16.79). The relative risks of warfarin (RR = 2.15, 95%CI: 1.43, 3.24), TIPS *plus* warfarin (RR = 7.83, 95%CI: 3.44, 17.80) and LMWH-warfarin sequential (RR = 2.26, 95%CI:1.16,4.42) were significantly favorable than that of control. TIPS *plus* warfarin was above heparin *plus* DOACs *plus* warfarin (RR = 3.69, 95%CI: 2.38, 5.71). The LMWH-DOACs sequential was likewise superior to the LMWH-warfarin sequential (RR = 3.50, 95%CI: 1.62, 7.57) in Figure 3. Comparison with control in network meta-analysis (Table 2), DOACs (RR = 2.15, 95%CI: 1.33, 3.48), LMWH (RR = 1.41, 95%CI: 1.01, 1.99), TIPS (RR = 5.68, 95%CI: 2.63, 12.24), warfarin (RR = 2.16, 95%CI: 1.46, 3.21), EBL *plus* propranolol (RR = 2.80, 95%CI: 1.18, 6.60), LMWH-DOACs sequential (RR = 7.92, 95%CI: 2.85, 21.99) and LMWH-warfarin sequential (RR = 2.26, 95%CI: 1.16, 4.42) significantly improved the incidence of complete recanalization. DOACs were statistically inferior to TIPS (RR = 0.38, 95%CI: 0.15, 0.94), TIPS *plus* warfarin (RR = 0.35, 95%CI: 0.14, 0.87) and LMWH-DOACs sequential (RR = 0.27, 95%CI: 0.09, 0.84). LMWH was inferior to TIPS (RR = 0.25, 95%CI: 0.11, 0.58), TIPS *plus* warfarin (RR = 0.23, 95%CI: 0.10, 0.54) and LMWH-DOACs sequential (RR = 0.18, 95%CI: 0.06, 0.52). TIPS was more favorable than warfarin (RR = 2.63, 95%CI: 1.11, 6.23), EBL *plus* propranolol (RR = 2.03, 95%CI: 1.38, 2.98) and heparin *plus* DOACs *plus* warfarin (RR = 3.71, 95%CI: 2.40, 5.71). Warfarin was below TIPS *plus* warfarin (RR = 0.36, 95%CI: 0.15, 0.84) and LMWH-DOACs sequential (RR = 0.27, 95%CI: 0.09, 0.82). EBL *plus* propranolol was beneath TIPS *plus* warfarin (RR = 0.46, 95%CI: 0.31, 0.68), but over heparin *plus* DOACs *plus* warfarin (RR = 1.83, 95%CI: 1.02, 3.25). LMWH-DOACs sequential was superior to LMWH-warfarin sequential (RR = 3.50, 95%CI: 1.62, 7.57) and heparin *plus* DOACs *plus* warfarin (RR = 5.17, 95%CI: 1.37, 19.49). The anticoagulation drugs were ranked based on their SUCRA values, with the LMWH-DOACs sequential (92.7%), TIPS *plus* warfarin (91.3%), and TIPS (80.3%) emerging as the top three effective treatments. Following these were EBL *plus* propranolol (55.0%), LMWH-warfarin sequential (47.0%), warfarin (44.9%), DOACs (44.5%), heparin *plus* DOACs *plus* warfarin (23.3%), and LMWH (18.7%). Control (2.4%) demonstrated the least effectiveness according to Figure 4.

**TABLE 2:** Results of network meta-analysis for complete and partial recanalization.

DOACs	NA	1.76 (0.16, 19.25)	2.73 (0.22, 33.72)	0.94 (0.07, 11.90)	3.39 (0.31, 37.31)	2.36 (0.19, 29.42)	4.72 (0.57, 38.87)	1.21 (0.12, 11.65)	6.81 (0.88, 52.73)	**2.83** **(1.84, 4.33)**
1.52 (0.91, 2.55)	LMWH	NA	NA	NA	NA	NA	NA	NA	NA	
**0.38 (0.15, 0.94)**	**0.25 (0.11, 0.58)**	TIPS	1.55 (0.23, 10.49)	0.54 (0.23, 1.24)	1.92 (0.98, 3.78)	1.34 (0.20, 9.18)	2.68 (0.70, 10.19)	0.68 (0.30, 1.54)	**3.86 (1.12, 13.30)**	
1.00 (0.59, 1.67)	0.65 (0.42, 1.02)	**2.63 (1.11, 6.23)**	Warfarin	0.35 (0.04, 2.81)	1.24 (0.18, 8.53)	0.87 (0.11, 6.92)	1.73 (0.37, 8.15)	0.44 (0.08, 2.57)	2.50 (0.58, 10.80)	
0.77 (0.29, 2.06)	0.51 (0.20, 1.27)	**2.03 (1.38, 2.98)**	0.77 (0.30, 1.99)	EBL plus Propranolol	**3.59 (1.22, 10.58)**	2.50 (0.31, 20.47)	**5.00 (1.03, 24.30)**	1.28 (0.40, 4.13)	**7.22 (1.62, 32.25)**	
**0.35 (0.14, 0.87)**	**0.23 (0.10, 0.54)**	0.93 (0.87, 1.01)	**0.36 (0.15, 0.84)**	**0.46 (0.31, 0.68)**	TIPS plus Warfarin	0.70 (0.10, 4.83)	1.39 (0.36, 5.38)	**0.36 (0.15, 0.82)**	2.01 (0.57, 7.04)	
**0.27 (0.09, 0.84)**	**0.18 (0.06, 0.52)**	0.72 (0.20, 2.57)	**0.27 (0.09, 0.82)**	0.35 (0.09, 1.34)	0.77 (0.21, 2.75)	LMWH-DOACs sequential	2.00 (0.50, 8.00)	0.51 (0.09, 3.00)	2.89 (0.66, 12.64)	
0.95 (0.42, 2.17)	0.62 (0.29, 1.32)	2.51 (0.91, 6.95)	0.95 (0.44, 2.08)	1.24 (0.42, 3.67)	2.69 (0.97, 7.43)	**3.50 (1.62, 7.57)**	LMWH-Warfarin sequential	**0.26 (0.08, 0.77)**	1.44 (0.87, 2.40)	
1.41 (0.53, 3.72)	0.92 (0.37, 2.30)	**3.71 (2.40, 5.71)**	1.41 (0.55, 3.59)	**1.83 (1.02, 3.25)**	**3.97 (2.58, 6.09)**	**5.17 (1.37, 19.49)**	1.48 (0.50, 4.35)	Heparin plus DOACs plus Warfarin	**5.65 (2.12, 15.05)**	
**2.15 (1.33, 3.48)**	**1.41 (1.01, 1.99)**	**5.68 (2.63, 12.24)**	**2.16 (1.46, 3.21)**	**2.80 (1.18, 6.60)**	**6.08 (2.83, 13.08)**	**7.92 (2.85, 21.99)**	**2.26 (1.16, 4.42)**	1.53 (0.66, 3.58)	Control	
**1.95 (1.37, 2.78)**										

Note: The results in the lower left section are the complete recanalization, and the results in the upper right section are the partial recanalization. Comparisons between treatments should be read from left to right and the estimate is in the cell in common between the upper-left-defining treatment and the lower-right-defining treatment. The relative risks (RR) greater than 1 favour the upper-left-defining treatment. To obtain RRs, for comparisons in the opposite direction, reciprocals should be taken. Significant results are in bold and underlined. TIPS, transjugular intrahepatic portal system shunt; EBL, endoscopic band ligation; DOACs, direct oral anticoagulants; LMWH, low molecular weight heparin; NA, not available.

#### 3.4.2 Partial recanalization

A total of 8 studies (Ai et al., 2020; Chung et al., 2014; Gao et al., 2023; Luo et al., 2015; Lv et al., 2018; Nagaoki et al., 2018; Wang et al., 2016; Zhang et al., 2023) with 951 patients had reported data on partial recanalization, and the corresponding network plots were depicted in Figure 2. Table 2 and Figure 3 presented the findings from the study regarding traditional paired and network meta-analysis of partial recanalization. The effect of active therapeutic strategies on increasing partial recanalization rate was elevated above that of control (RR = 2.83, 95%CI: 1.84, 4.33). TIPS *plus* warfarin was inferior to heparin *plus* DOACs *plus* warfarin in direct comparisons (RR = 0.29, 95%CI: 0.12, 0.75), and heparin *plus* DOACs *plus* warfarin was superior to control (RR = 5.65, 95%CI: 2.12, 15.05) in Figure 3. In comparisons of network meta-analysis (Table 2), TIPS (RR = 3.86, 95%CI: 1.12, 13.30), EBL *plus* propranolol (RR = 7.22, 95%CI: 1.62, 32.25) and heparin *plus* DOACs *plus* warfarin (RR = 5.65, 95%CI: 2.12, 15.05) were elevated above control. EBL *plus* propranolol was found to be associated with elevated levels of TIPS *plus* warfarin (RR = 3.59, 95%CI: 1.22, 10.58) and LMWH-warfarin sequential (RR = 5.00, 95%CI: 1.03, 24.30). TIPS *plus* warfarin (RR = 0.36, 95%CI: 0.15, 0.82) and LMWH-warfarin sequential (RR = 0.26, 95%CI: 0.08, 0.77) were inferior to heparin *plus* DOACs *plus* warfarin. The most effective treatment for active therapeutic strategies in order of SUCRA was EBL *plus* propranolol (84.2%), heparin *plus* DOACs *plus* warfarin (77.3%), DOACs (75.2%), followed by TIPS (58.7%), LMWH-DOACs sequential (49.6%), warfarin (43.8%), TIPS *plus* warfarin (32.2%), and LMWH-warfarin sequential (22.8%). The poorest treatment effect was control (6.2%) (Figure 4).

### 3.5 Secondary outcomes

#### 3.5.1 Bleeding

A total of 11 studies (Ai et al., 2020; Florescu et al., 2021; Gao et al., 2023; Lv et al., 2021; Lv et al., 2018; Nagaoki et al., 2018; Pettinari et al., 2019; Senzolo et al., 2012; Wang et al., 2016; Zhang et al., 2023; Zhou et al., 2020) with 1,275 patients provided data on bleeding, and the corresponding network plots were depicted in Supplementary Figure S1. Supplementary Table S3 presented the findings from the study regarding traditional paired and network meta-analysis of bleeding events. There was no significant difference in bleeding reduction between the active therapeutic strategies and control (RR = 1.02, 95%CI: 0.78, 1.33). In direct comparisons, TIPS was weaker than EBL *plus* propranolol (RR = 0.35, 95%CI: 0.15, 0.81), TIPS *plus* warfarin (RR = 0.48, 95%CI: 0.27, 0.84) and heparin *plus* DOACs *plus* warfarin (RR = 0.41, 95%CI: 0.21, 0.81). In comparisons of network meta-analysis, there were no statistical differences among DOACs, LMWH, TIPS, EBL *plus* propranolol, LMWH *plus* warfarin, TIPS *plus* warfarin, LMWH-DOACs sequential, LMWH-warfarin sequential, heparin *plus* DOACs *plus* warfarin and control. Active therapeutic strategies were ranked by SUCRA, with the top three being TIPS (87.5%), LMWH (77.5%), and control (52%), followed by LMWH-warfarin sequential (50.1%), LMWH *plus* warfarin (48.8%), TIPS *plus* warfarin (48.8%), LMWH *plus* DOACs *plus* warfarin (48%), EBL *plus* propranolol (39.7%), and LMWH-DOACs sequential (25.8%). The treatment was least effective with DOACs (22%) (Figure 4).

#### 3.5.2 Major bleeding

A total of 6 studies (Chung et al., 2014; Florescu et al., 2021; Joseph and Rejeski, 2020; Lv et al., 2021; Naymagon et al., 2021; Zhang et al., 2023) with 1,051 patients reported data on hemorrhage, and the corresponding network diagram was presented in Supplementary Figure S2. Supplementary Table S4 presented the results of study regarding traditional paired and network meta-analysis of significant bleeding. There was no significant difference in the reduction of major bleeding between the active therapeutic strategies and control (RR = 0.79, 95%CI: 0.57, 1.10). Regardless of direct or network meta-analysis, all results were no statistical differences. The most effective drugs were found to be LMWH *plus* warfarin (85.4%), DOACs (77.9%), and TIPS (63.1%). Following closely were TIPS *plus* warfarin (50.4%) and warfarin alone (47.6%), control (28.5%), LMWH (24%). The least effective treatment was observed with heparin *plus* DOACs *plus* warfarin at 23.1% (Figure 4).

#### 3.5.3 Mortality

A total of 9 studies (Chung et al., 2014; Joseph and Rejeski, 2020; Luo et al., 2015; Lv et al., 2021; Lv et al., 2018; Naymagon et al., 2021; Senzolo et al., 2012; Wang et al., 2016; Zhang et al., 2023) with 1,226 patients reported data on mortality, and the correlation network plot was depicted in Supplementary Figure S3. Supplementary Table S5 presented the results of the study regarding traditional paired and network meta-analysis of mortality. In terms of mortality, active therapeutic strategies was inferior to control (RR = 0.62, 95%CI: 0.48, 0.81). In direct comparisons, warfarin (RR = 0.41, 95%CI: 0.17, 0.98) and heparin *plus* DOACs *plus* warfarin (RR = 0.46, 95%CI: 0.23, 0.92) were less than control. Comparisons of network meta-analysis, all results were no statistical differences. The three most effective drugs were warfarin (77.8%), heparin *plus* DOACs *plus* warfarin (74.1%), and DOACs (67%), followed by LMWH (60.9%), TIPS *plus* warfarin (38.7%), TIPS (31.5%) and EBL *plus* propranolol (30.3%). The least effective treatment was control with an effectiveness rate of 19.7% (Figure 4).

#### 3.5.4 PVT extension

A total of 11 studies (Ai et al., 2020; Chen et al., 2016; Chung et al., 2014; Gao et al., 2023; Lv et al., 2021; Lv et al., 2018; Nagaoki et al., 2018; Naymagon et al., 2021; Senzolo et al., 2012; Zhang et al., 2023; Zhou et al., 2020) with 1,228 patients reported data on PVT extensions, and the associated network plot were shown in Supplementary Figure S4. Supplementary Table S6 presented the results of traditional paired and network meta-analysis in terms of the incidence of PVT extensions. In terms of the incidence of PVT expansion, active therapeutic strategies were superior to control (RR = 0.44, 95%CI: 0.32, 0.60). In the direct comparisons, LMWH (RR = 0.21, 95%CI: 0.09, 0.50) and LMWH-warfarin sequential (RR = 0.40, 95%CI: 0.21, 0.77) were superior to control. TIPS *plus* warfarin was superior to heparin *plus* DOACs *plus* warfarin (RR = 0.04, 95%CI: 0.00, 0.81). LMWH-DOACs sequential was superior to LMWH-warfarin sequential (RR = 0.11, 95%CI: 0.02, 0.75). Comparisons of network meta-analysis showed that DOACs were inferior to LMWH-DOACs sequential (RR = 17.05, 95%CI: 1.40, 208.48). LMWH *plus* warfarin was inferior to TIPS *plus* warfarin (RR = 24.65, 95%CI: 1.04, 581.70) and LMWH-DOACs sequential (RR = 17.19, 95%CI: 1.76, 168.29). The LMWH-DOACs sequential was superior to the LMWH-warfarin sequential (RR = 0.11, 95%CI: 0.02, 0.75), heparin *plus* DOACs *plus* warfarin (RR = 0.09, 95%CI: 0.01, 0.91) and LMWH *plus* DOACs *plus* warfarin (RR = 0.07, 95%CI: 0.01, 0.62). LMWH (RR = 0.21, 95%CI: 0.09, 0.50), TIPS *plus* warfarin (RR = 0.03, 95%CI: 0.00, 0.60), LMWH-DOACs sequential (RR = 0.04, 95%CI: 0.01, 0.33) and LMWH-warfarin sequential (RR = 0.40, 95%CI: 0.21, 0.77) were superior to control. Active therapeutic strategies were ranked by SUCRA and the upper three with better effects were TIPS *plus* warfarin (88.8%), LMWH-DOACs sequential (87.5%), TIPS (79%), followed by LMWH (67.2%), warfarin (51.2%), LMWH-warfarin sequential (47%), EBL *plus* propranolol (46.9%), heparin *plus* DOACs *plus* warfarin (40%), heparin *plus* DOACs *plus* warfarin (32.1%), DOACs (25.9%), LMWH *plus* warfarin (24%). The most poorly treated was control (10.5%) (Figure 4).

#### 3.5.5 Hepatic encephalopathy

A total of four studies (Luo et al., 2015; Lv et al., 2021; Lv et al., 2018; Wang et al., 2016) with 581 patients provided data on hepatic encephalopathy, and the corresponding network plots were illustrated in Supplementary Figure S5. Supplementary Table S7 presented the findings of the study regarding traditional paired and network meta-analysis of hepatic encephalopathy. In terms of the incidence of hepatic encephalopathy, the active therapeutic strategies was inferior to control (RR = 2.54, 95%CI: 1.39, 4.66). In direct comparisons, TIPS was inferior to heparin *plus* DOACs *plus* warfarin (RR = 8.23, 95%CI: 2.01, 33.66) and control (RR = 3.14, 95%CI: 1.15, 8.54). TIPS *plus* warfarin was inferior to heparin *plus* DOACs *plus* warfarin (RR = 9.43, 95%CI: 2.37, 37.51) and control (RR = 3.59, 95%CI: 1.37, 9.41). In comparisons of network meta-analysis, TIPS (RR = 8.19, 95%CI: 2.01, 33.32), EBL *plus* propranolol (RR = 8.14, 95%CI: 1.84, 35.91) and TIPS *plus* warfarin (RR = 9.45, 95%CI: 2.38, 37.56) were inferior to heparin *plus* DOACs *plus* warfarin. TIPS (RR = 3.12, 95%CI: 1.15, 8.44), EBL *plus* propranolol (RR = 3.10, 95%CI: 1.03, 9.37) and TIPS *plus* warfarin (RR = 3.60, 95%CI: 1.38, 9.42) was inferior to control. The most treatment effective of active therapeutic strategies in order of SUCRA was heparin *plus* DOACs *plus* warfarin (96.4%), followed by control (77.3%), TIPS (33.1%) and EBL *plus* propranolol (29%). The most inferior treatment effect was TIPS *plus* warfarin (14.1%) (Figure 4).

### 3.6 Test of inconsistency

The use of each network plot allowed for the assessment of inconsistency by forming a closed loop in indirect comparisons of all outcomes. Inconsistencies between the estimates from direct analysis and network meta-analysis were examined using node-splitting, as detailed in Supplementary Tables S8–S14 All outcomes, including complete recanalization, partial recanalization, bleeding, major bleeding, mortality, PVT expansion, and hepatic encephalopathy showed no significant association (P> 0.05), indicating absence of inconsistency.

### 3.7 Sensitivity analysis

For the included articles in this network meta-analysis, we removed data from studies with Child-Pugh C >20% of the enrolled population to test the robustness for all outcomes. Pooled data showed no substantial change in outcomes when enrollment was changed. Sensitivity analyses of all outcomes were shown in Supplementary Tables S15–S21.

### 3.8 Publication bias

The funnel plots for complete recanalization, partial recanalization, hemorrhage, massive hemorrhage, mortality, PVT expansion, and hepatic encephalopathy were illustrated in Supplementary Figures S6–S12. Visual examination was conducted based on the symmetry criterion revealing a concentration of scatter points along the central axis. The symmetrical distribution of these points in the funnel plot suggested minimal impact from publication bias on each outcome.

## 4 Discussion

The increased occurrence of PVT in patients with cirrhosis can be attributed to multiple factors including vascular rupture and alterations in blood flow velocity, especially during the decompensated phase (Zhao et al., 2020). Nevertheless, there seemed to be no direct association between PVT and prognostic outcomes like gastrointestinal bleeding, liver function deterioration or mortality in cirrhotic patients (Hayashi et al., 2019). Notably though, PVT frequently coincided with the onset or exacerbation of ascites and/or hepatic encephalopathy which significantly worsened overall patient prognosis (Rodriguez-Castro et al., 2019). Therefore, the prevention of PVT remained imperative in patients with cirrhosis. However, the implementation of therapeutic strategies in this patient population had encountered certain challenges primarily attributed to the altered hemostatic mechanism observed in individuals with cirrhosis (Tantai et al., 2019). Compared to other patients, those with liver cirrhosis exhibited diminished levels of coagulation factors due to hepatic lesions, rendering them more susceptible to bleeding complications. Consequently, it was essential to develop additional protocols for administering anticoagulation in patients with cirrhosis. The previous systematic reviewed solely assessed the effectiveness and safety of anticoagulation treatment in patients with PVT in cirrhosis, without providing corresponding recommendations for anticoagulation treatment (Huang et al., 2020). This study employed a network format meta-analysis to comprehensively evaluate the efficacy and safety of various anticoagulation regimens in liver cirrhosis with PVT, aiming to establish reliable treatment strategies for clinical practice. The sequential administration of LMWH-DOACs demonstrated a significant augmentation in the occurrence of complete recanalization events. The combined therapy involving EBL and propranolol exhibited an elevated frequency of partial recanalization events. Conversely, TIPS displayed a remarkable decrease in bleeding occurrences. Moreover, when used together, LMWH and warfarin led to a diminished prevalence of major bleeding incidents; however, warfarin alone exhibited superior efficacy in mitigating mortality cases. Remarkably, the utilization of TIPS alongside warfarin indicated lowered frequencies regarding PVT expansion and hepatic encephalopathy. Furthermore, all therapeutic protocols showcased equivalent effectiveness concerning hemorrhage reduction as well as mitigation against severe bleedings or fatalities.

Although self-recanalization may occur in some patients with cirrhosis, study had demonstrated the efficacy of anticoagulant therapy in improving recanalization rates (Rodrigues et al., 2019). These findings were consistent with similar conclusions drawn from research. In one study, anticoagulation was found to increase recanalization by a factor of 3.5, regardless of the severity of PVT (Zhang et al., 2021). Therefore, despite the possibility of self-recanalization in cirrhotic patients presenting with thrombosis, observation is not recommended while anticoagulation is strongly advised. On the contrary, there seem to exist a therapeutic time window for anticoagulation treatment. There was a study had demonstrated that early initiation of anticoagulation treatment can significantly enhance the recanalization rate in comparison to delayed anticoagulation treatment. The implementation of anticoagulation within 2 weeks–6 months, as opposed to no or delayed anticoagulation treatment, can effectively ameliorate the recanalization rate (Ghazaleh et al., 2021).

In this study, the sequential administration of LMWH-DOACs demonstrated the highest efficacy in increasing the rate of complete recanalization, followed by TIPS combined with warfarin and standalone TIPS. Conversely, EBL in combination with propranolol exhibited superior effectiveness in enhancing the partial recanalization rate, followed by heparin combined with DOACs and warfarin as well as standalone DOACs. Control did not yield any improvement in either complete or partial recanalization rates. The optimal therapeutic regimen for improving recanalization rates was specifically the sequential administration of LMWH and edoxaban. In patients with cirrhosis, edoxaban offered distinct advantages as an anticoagulant due to its hepatic metabolism-independent nature. Furthermore, evidence suggested that edoxaban may mitigate the risk of bleeding in patients with severe thromboembolism and could be considered for treating individuals with severe PVT (Hanafy et al., 2023). However, further empirical verification is required to determine the exact efficacy and safety of edoxaban. Network Meta-analysis results indicated no disparity in complete recanalization rates between TIPS combined with anticoagulant therapy and TIPS alone. Similar conclusions had been drawn from other relevant studies. It should be noted that the administration of anticoagulants before or after TIPS surgery did not appear to significantly impact PVT recanalization rates (Wu et al., 2022).

One of the major complications in patients with liver cirrhosis was gastrointestinal bleeding, which can be attributed to various predisposing factors such as thrombocytopenia, decreased synthesis of coagulation factors, and secondary hyperfibrinolysis (Serper et al., 2021). Therefore, it was imperative for individuals with PVT in cirrhosis to remain vigilant regarding potential bleeding complications during anticoagulation treatment. However, no significant increase in bleeding events associated with anticoagulation treatment was observed. In this study, none of the regimens, including control, demonstrated an increased incidence of bleeding events. There was a study had reached similar conclusions; thus, therapeutic strategies appeared to be relatively safe for patients with cirrhosis (Scheiner et al., 2018). Previous research had consistently indicated that variceal rupture was the primary cause of bleeding in most cirrhotic patients, attributed to portal hypertension, while anticoagulation treatment exhibited limited impact on bleeding (Zhang et al., 2021). The ACG clinical guidelines also confirmed that the presence of gastroesophageal varices did not constitute a contraindication to anticoagulation treatment (Simonetto et al., 2020). However, it is imperative to ensure adequate primary or secondary prevention of variceal bleeding prior to initiating anticoagulation treatment in order to effectively mitigate the impact of portal hypertension on treatment outcomes.

In terms of reducing the incidence of bleeding events, TIPS demonstrated the highest efficacy, followed by LMWH and control, while DOACs exhibited the least effectiveness. In relation to decreasing the rate of major bleeding, LMWH combined with warfarin displayed superior efficacy, followed by DOACs and TIPS; whereas heparin in combination with DOACs and warfarin showed the lowest effectiveness. The latest study (Giri et al., 2023) conducted demonstrated that the utilization of an 8 mm stent has a significant impact on preventing bleeding and is equally recommendable for patients with cirrhosis. This study arrived at a similar conclusion, highlighting TIPS as the most effective approach in reducing bleeding incidents. However, both LMWH and DOACs appeared to be more efficacious than TIPS in managing major bleeding events. Data analysis revealed a higher occurrence of bleeding events among patients receiving warfarin AC. It is recommended that clinicians utilize LMWH or DOACs instead of warfarin to mitigate the incidence of bleeding events in order to address the challenge posed by unpredictable bleeding events in cirrhotic patients, attributed to rebalanced hemostasis. This altered equilibrium between procoagulant and anticoagulant factors within the plasma of cirrhotic individuals gives rise to this distinctive hemostatic characteristic (Pan et al., 2022). Further exploration of treatment options is warranted for effectively reducing bleeding events in PVT in cirrhosis.

In patients with liver cirrhosis, the occurrence of PVT was not associated with the progression of hepatic decompensation or increased mortality due to cirrhosis. However, it may potentially impact post-liver transplantation survival. Therefore, mortality was a significant concern in anticoagulation treatment. In this study, none of the therapeutic option demonstrated an increased incidence of mortality. Consequently, therapeutic strategies exhibited a favorable safety profile in patients with cirrhosis. According to previous studies, there was no significant concern regarding mortality risk in patients with PVT in cirrhosis when undergoing anticoagulation therapy, although the possibility of bleeding exists (Guo et al., 2022). One study reported that anticoagulation treatment was administered to patients with Child-Pugh class A or B cirrhosis and they were followed up for a period of 6 months without any fatal bleeding incidents (Noronha Ferreira et al., 2019a). Further studies demonstrated no significant association between anticoagulation treatment and mortality at 1 or 2 years of follow-up. However, in the study conducted after 3 years of follow-up, anticoagulation treatment exhibited a statistically significant reduction in mortality. This suggests a potential delayed effect of anticoagulation treatment on reducing mortality. However, study (Joseph and Rejeski, 2020) may have affected the stability of the pooled results when it was used to reduce mortality. Consequently, the outcome of death in this study was more sensitive, further data with long-term follow-up are needed.

DOACs offered distinct advantages in the treatment of PVT in cirrhosis. One significant factor that attracts cirrhotic patients to DOACs was their superior efficacy. Compared to LMWH, DOACs provided a more convenient administration route by eliminating the need for subcutaneous injections. Unlike warfarin, DOACs obviated the necessity for therapeutic drug monitoring and facilitate easier assessment of adequate treatment for cirrhotic patients with prothrombin disorders. Furthermore, DOACs exhibited more predictable pharmacokinetic and pharmacodynamic properties while being less susceptible to drug interactions and dietary influences than warfarin (Vranckx et al., 2018). Additionally, DOACs demonstrateed a more rapid onset of action in comparison to warfarin, thereby eliminating the need for induction and bridging therapy. Unlike LMWH, which had a shorter duration of effect and limited availability of reversal agents, DOACs required 24–72 h for *in vivo* metabolism and can be discontinued 24–72 h prior to surgery in order to minimize their impact on post-transplant survival.

In the study conducted by Ghazaleh et al. (2021), oral anticoagulation agents demonstrated efficacy in increasing the rate of portal vein recanalization, with edoxaban exhibiting the highest effectiveness. Among all anticoagulation agents, warfarin was found to be the least effective. The study conducted by Li et al. (2023) suggested that anticoagulation treatment did not pose a bleeding risk and was relatively safe for patients with PVT in cirrhosis. Compared to DOACs, traditional anticoagulation drugs such as LMWH or warfarin were shown to reduce the incidence of bleeding events. It was important to note that bleeding risk is associated with factors such as age, duration of treatment, and Child-Pugh grade; therefore, clinicians should exercise extra caution when dealing with high-risk patients. DOACs were not recommended for older adults and patients with Child-Pugh grade C. While some studies (Gao et al., 2023; Nagaoki et al., 2018; Naymagon et al., 2021; Senzolo et al., 2012) had combined TIPS with anticoagulation treatment as intervention measures, Guo et al.’s study (Guo et al., 2022) indicated that there was no significant difference in efficacy between TIPS combined with anticoagulation treatment and anticoagulation treatment alone for patients with PVT in cirrhosis. Furthermore, the potential of TIPS in the treatment of PVT in cirrhosis requires further exploration. A meta-analysis conducted by Zhang et al. (2023) revealed that anticoagulation agents were associated with increased rates of PVT recanalization, prolonged and shortened PVT, independent of bleeding incidence including major bleeding and variceal bleeding. Additionally, DOACs demonstrated higher rates of PVT recanalization compared to warfarin.

The present study still has certain limitations. Firstly, the inclusion of various stent techniques, types, and manufacturers may have introduced significant heterogeneity into this meta-analysis; however, these factors were not accounted for in our analysis. Nevertheless, a substantial portion of the observed heterogeneity can be attributed to random effects models. Additionally, anticoagulation pharmacotherapy may lead to increased PT and INR levels, which are components of the Child-Pugh score and MELD score respectively. This could potentially result in an underestimation of liver function. However, it is important to acknowledge that this represents an unavoidable confounding factor when assessing liver function. Therefore, further advancements in data analysis techniques are necessary to mitigate the impact of confounders on study outcomes. Thirdly, the majority of studies included a follow-up period of 6 months. However, it is crucial to conduct longer-term follow-ups to accurately evaluate the impact of anticoagulation treatment on recanalization rates and mortality among cirrhotic patients with PVT. Moreover, fourthly, there appears to be a potential differential effect of anticoagulation treatment based on the Child-Pugh score; nevertheless, subgroup analyses were not feasible due to missing data. Therefore, further comprehensive data are required for future in-depth investigations.

## 5 Conclusion

In this study, active anticoagulants were recommended for cirrhosis with PVT. The TIPS *plus* warfarin, LMWH-DOACs sequential, and TIPS improved the complete recanalization rate most effectively, and the EBL *plus* propranolol, heparin *plus* DOACs *plus* warfarin, and DOACs were highly recommended for increasing the incidence of partial recanalization. Warfarin and TIPS were recommended for reducing the frequency of bleeding events, while LMWH *plus* warfarin and DOACs proved to be most effective in decreasing the rate of major bleeding events. Warfarin, heparin *plus* DOACs *plus* warfarin, and DOACs demonstrated the most significant reduction in mortality rates, highlighting its potential as an effective intervention. TIPS *plus* warfarin, LMWH-DOACs sequential, and TIPS were recommended for reducing the occurrence of PVT expansion. Heparin *plus* DOACs *plus* warfarin was recommended for reducing the occurrence of hepatic encephalopathy, and protocols that involve TIPS were generally associated with a higher risk of hepatic encephalopathy. However, a longer follow-up period is necessary to comprehensively evaluate the efficacy of active anticoagulants therapy in patients with PVT in cirrhosis. Further studies are warranted to obtain additional experimental data for establishing a more comprehensive treatment plan through meticulous data analysis.

## Data Availability

The original contributions presented in the study are included in the article/Supplementary Material, further inquiries can be directed to the corresponding authors.
