# Supplementary material for: Administration of anticoagulation strategies for portal vein thrombosis in cirrhosis: network meta-analysis
Source: Front Pharmacol. 2025 Jan 6;15:1462338. doi: 10.3389/fphar.2024.1462338 (PMC11743941; doi:10.3389/fphar.2024.1462338)

**Supplementary Material**

**Supplementary Table 1** Risk of bias for randomized controlled trials
**Supplementary Table 2** Risk of bias for non-randomized controlled trials **Supplementary Table 3** Results of traditional paired and network meta-analysis for bleeding

**Supplementary Table 4** Results of traditional paired and network meta-analysis for major bleeding

**Supplementary Table 5** Results of traditional paired and network meta-analysis for mortality

**Supplementary Table 6** Results of traditional paired and network meta-analysis for PVT extension

**Supplementary Table 7** Results of traditional paired and network meta-analysis for hepatic encephalopathy

**Supplementary Table 8** Test of inconsistency for complete recanalization
**Supplementary Table 9** Test of inconsistency for partial recanalization

**Supplementary Table 10** Test of inconsistency for bleeding
**Supplementary Table 11** Test of inconsistency for major bleeding
**Supplementary Table 12** Test of inconsistency for mortality
**Supplementary Table 13** Test of inconsistency for PVT extension

**Supplementary Table 14** Test of inconsistency for hepatic encephalopathy

**Supplementary Figure 1** Network plot for bleeding

**Supplementary Figure 2** Network plot for major bleeding

**Supplementary Figure 3** Network plot for mortality

**Supplementary Figure 4** Network plot for PVT extension

**Supplementary Figure 5** Network plot for hepatic encephalopathy

**Supplementary Figure 6** Funnel plot for complete recanalization
**Supplementary Figure 7** Funnel plot for partial recanalization
**Supplementary Figure 8** Funnel plot for bleeding
**Supplementary Figure 9** Funnel plot for major bleeding
**Supplementary Figure 10** Funnel plot for mortality **Supplementary Figure 11** Funnel plot for PVT extension

**Supplementary Figure 12** Funnel plot for hepatic encephalopathy

**Supplementary Table 1** Risk of bias for randomized controlled trials

| **Study** | **Year** | **Bias arising from the randomization process** | **Bias due to deviations from intended interventions** | **Bias due to missing outcome data** | **Bias in measurement of the outcome** | **Bias in selection of the reported result** | **Overall risk of bias** |
| --- | --- | --- | --- | --- | --- | --- | --- |
| Luo | 2015 | Low risk | Low risk | Low risk | Low risk | Low risk | Low risk |
| Wang | 2016 | Low risk | Low risk | Low risk | Low risk | Low risk | Low risk |
| Lv | 2018 | Low risk | Low risk | Low risk | Low risk | Low risk | Low risk |
| Zhou | 2020 | Low risk | Low risk | Low risk | Some concerns | Low risk | Moderate risk |
| Gao | 2023 | Low risk | Low risk | Low risk | Low risk | Low risk | Low risk |

**Supplementary Table 2** Risk of bias for non-randomized controlled trials

| **Study** | **Year** | **Bias due to confounding** | **Bias in selection of participants into the study** | **Bias in classification of interventions** | **Bias due to deviations from intended interventions** | **Bias due to missing data** | **Bias in measurement of outcomes** | **Bias in selection of the reported result** |
| --- | --- | --- | --- | --- | --- | --- | --- | --- |
| Garcovich | 2011 | Low risk | Low risk | Low risk | Low risk | Low risk | Low risk | Serious risk |
| Senzolo | 2012 | Moderate risk | Low risk | Low risk | Moderate risk | Low risk | Low risk | Moderate risk |
| Caracciolo | 2013 | Moderate risk | Low risk | Low risk | Low risk | Serious risk | Low risk | Serious risk |
| Chung | 2014 | Moderate risk | Low risk | Low risk | Moderate risk | Low risk | Low risk | Moderate risk |
| Chen | 2016 | Moderate risk | Low risk | Low risk | Low risk | Moderate risk | Low risk | Moderate risk |
| Nagaoki | 2018 | Low risk | Low risk | Low risk | Low risk | Low risk | Low risk | Moderate risk |
| Noronha | 2019 | Moderate risk | Low risk | Low risk | Low risk | Low risk | Low risk | Moderate risk |
| Pettinar | 2019 | Low risk | Low risk | Low risk | Low risk | Low risk | Low risk | Moderate risk |
| Ai | 2020 | Moderate risk | Low risk | Low risk | Low risk | Low risk | Low risk | Moderate risk |
| Joseph | 2020 | Moderate risk | Low risk | Low risk | Low risk | Low risk | Low risk | Moderate risk |
| Lv | 2021 | Moderate risk | Low risk | Low risk | Moderate risk | Low risk | Low risk | Moderate risk |
| Florescu | 2021 | Low risk | Low risk | Low risk | Low risk | Low risk | Low risk | Moderate risk |
| Zhang | 2023 | Moderate risk | Low risk | Low risk | Low risk | Low risk | Low risk | Moderate risk |

**Supplementary Table 3** Results of traditional paired and network meta-analysis for bleeding

| **DOACs** | NA | NA | NA | NA | NA | NA | NA | NA | 3.00  (0.33, 27.63) | 1.02  (0.78, 1.33) |
| --- | --- | --- | --- | --- | --- | --- | --- | --- | --- | --- |
| 5.88  (0.35, 98.16) | **LMWH** | NA | NA | NA | NA | NA | NA | NA | 0.51  (0.15, 1.68) |  |
| 6.45  (0.47, 88.58) | 1.10  (0.17, 6.88) | **TIPS** | **0.35**  **(0.15, 0.81)** | NA | **0.48**  **(0.27, 0.84)** | NA | NA | **0.41**  **(0.21, 0.81)** | 0.67  (0.30, 1.50) |  |
| 2.24  (0.12, 40.36) | 0.38  (0.04, 3.46) | 0.35  (0.10, 1.18) | **EBL *plus* Propranolol** | NA | NA | NA | NA | NA | NA |  |
| 2.78  (0.22, 34.70) | 0.47  (0.09, 2.58) | 0.43  (0.11, 1.65) | 1.24  (0.20, 7.64) | **LMWH *plus* Warfarin** | NA | NA | NA | NA | 1.02  (0.34, 3.07) |  |
| 2.76  (0.21, 36.71) | 0.47  (0.08, 2.81) | 0.43  (0.17, 1.10) | 1.23  (0.26, 5.77) | 0.99  (0.27, 3.63) | **TIPS *plus* Warfari**n | NA | NA | 0.77  (0.49, 1.22) | 1.25  (0.66, 2.36) |  |
| 1.29  (0.05, 34.19) | 0.22  (0.01, 3.24) | 0.20  (0.02, 2.43) | 0.58  (0.04, 9.29) | 0.46  (0.04, 5.09) | 0.47  (0.04, 5.38) | **LMWH-DOACs sequential** | 2.25  (0.41, 12.28) | NA | NA |  |
| 2.90  (0.20, 41.48) | 0.49  (0.07, 3.28) | 0.45  (0.09, 2.24) | 1.29  (0.17, 9.74) | 1.04  (0.25, 4.40) | 1.05  (0.23, 4.80) | 2.25  (0.33, 15.28) | **LMWH-Warfarin sequential** | NA | 0.97  (0.42, 2.20) |  |
| 2.75  (0.22, 35.13) | 0.47  (0.08, 2.64) | 0.43  (0.15, 1.17) | 1.23  (0.25, 6.02) | 0.99  (0.30, 3.27) | 1.00  (0.38, 2.62) | 2.14  (0.19, 24.16) | 0.95  (0.21, 4.21) | **Heparin *plus* DOACs *plus* Warfarin** | 1.08  (0.43, 2.72) |  |
| 2.99  (0.27, 32.64) | 0.51  (0.11, 2.26) | 0.46  (0.16, 1.36) | 1.34  (0.26, 6.82) | 1.08  (0.48, 2.44) | 1.08  (0.40, 2.93) | 2.32  (0.25, 21.93) | 1.03  (0.32, 3.33) | 1.09  (0.45, 2.63) | **Control** | |

**Note:** The results in the lower left section are network meta-analysis, and the results in the upper right section are traditional paired meta-analysis. Comparisons between treatments should be read from left to right and the estimate is in the cell in common between the upper-left-defining treatment and the lower-right-defining treatment. The relative risks (RR) greater than 1 favour the lower-right-defining treatment. To obtain RRs for comparisons in the opposite direction, reciprocals should be taken. Significant results are in bold and underlined. TIPS: transjugular intrahepatic portal system shunt, EBL: endoscopic band ligation, DOACs: direct oral anticoagulants, LMWH: low molecular weight heparin, NA: not available.

**Supplementary Table 4** Results of traditional paired and network meta-analysis for major bleeding

| **DOACs** | 0.29  (0.04, 2.16) | NA | 0.37  (0.07, 2.05) | NA | NA | NA | 0.32  (0.05, 2.24) | 0.79  (0.57, 1.10) |
| --- | --- | --- | --- | --- | --- | --- | --- | --- |
| 0.29  (0.05, 1.63) | **LMWH** | NA | 1.24  (0.41, 3.70) | NA | NA | NA | 1.10  (0.53, 2.28) |  |
| 0.49  (0.08, 3.04) | 1.68  (0.57, 4.94) | **TIPS** | NA | NA | 0.82  (0.43, 1.56) | 0.56  (0.27, 1.16) | 0.67  (0.30, 1.50) |  |
| 0.41  (0.07, 2.20) | 1.40  (0.49, 3.98) | 0.83  (0.25, 2.78) | **Warfarin** | NA | NA | NA | 0.72  (0.29, 1.77) |  |
| 2.38  (0.08, 68.34) | 8.21  (0.40, 167.29) | 4.88  (0.24, 101.15) | 5.86  (0.27, 125.17) | **LMWH *plus* Warfarin** | NA | NA | 0.13  (0.01, 2.44) |  |
| 0.40  (0.07, 2.36) | 1.38  (0.52, 3.68) | 0.82  (0.43, 1.56) | 0.98  (0.32, 3.02) | 0.17  (0.01, 3.37) | **TIPS *plus* Warfarin** | 0.69  (0.39, 1.21) | 0.81  (0.41, 1.59) |  |
| 0.28  (0.05, 1.69) | 0.97  (0.35, 2.68) | 0.58  (0.28, 1.18) | 0.69  (0.22, 2.19) | 0.12  (0.01, 2.40) | 0.70  (0.40, 1.23) | **Heparin *plus* DOACs *plus* Warfarin** | 1.09  (0.54, 2.21) |  |
| 0.31  (0.06, 1.62) | 1.08  (0.52, 2.23) | 0.64  (0.29, 1.41) | 0.77  (0.31, 1.90) | 0.13  (0.01, 2.44) | 0.78  (0.40, 1.51) | 1.11  (0.55, 2.25) | **Control** | |

**Note:** The results in the lower left section are network meta-analysis, and the results in the upper right section are traditional paired meta-analysis. Comparisons between treatments should be read from left to right and the estimate is in the cell in common between the upper-left-defining treatment and the lower-right-defining treatment. The relative risks (RR) greater than 1 favour the lower-right-defining treatment. To obtain RRs for comparisons in the opposite direction, reciprocals should be taken. Significant results are in bold and underlined. TIPS: transjugular intrahepatic portal system shunt, EBL: endoscopic band ligation, DOACs: direct oral anticoagulants, LMWH: low molecular weight heparin, NA: not available.

**Supplementary Table 5** Results of traditional paired and network meta-analysis for mortality

| **DOACs** | 0.88  (0.26, 2.92) | NA | 1.09  (0.41, 2.87) | NA | NA | NA | 0.56  (0.19, 1.63) | **0.62**  **(0.48, 0.81)** |
| --- | --- | --- | --- | --- | --- | --- | --- | --- |
| 0.88  (0.28, 2.82) | **LMWH** | NA | 1.24  (0.41, 3.70) | NA | NA | NA | 0.61  (0.32, 1.14) |  |
| 0.58  (0.15, 2.20) | 0.66  (0.21, 2.08) | **TIPS** | NA | 0.95  (0.58, 1.55) | 1.91  (0.95, 3.82) | 1.08  (0.72, 1.63) | 0.82  (0.48, 1.38) |  |
| 1.18  (0.42, 3.29) | 1.33  (0.45, 3.93) | 2.03  (0.57, 7.19) | **Warfarin** | NA | NA | NA | **0.41**  **(0.17, 0.98)** |  |
| 0.57  (0.13, 2.42) | 0.64  (0.18, 2.33) | 0.97  (0.46, 2.06) | 0.48  (0.12, 1.92) | **EBL *plus* Propranolol** | NA | NA | NA |  |
| 1.07  (0.27, 4.31) | 1.21  (0.36, 4.11) | 1.84  (0.77, 4.42) | 0.91  (0.24, 3.43) | 1.90  (0.60, 5.99) | **Heparin *plus* DOACs *plus* Warfarin** | 0.55  (0.29, 1.06) | **0.46**  **(0.23, 0.92)** |  |
| 0.64  (0.18, 2.31) | 0.73  (0.24, 2.17) | 1.10  (0.54, 2.24) | 0.54  (0.16, 1.82) | 1.14  (0.42, 3.04) | 0.60  (0.26, 1.40) | **TIPS *plus* Warfarin** | 0.78  (0.49, 1.24) |  |
| 0.51  (0.18, 1.46) | 0.57  (0.25, 1.30) | 0.87  (0.40, 1.90) | 0.43  (0.16, 1.13) | 0.90  (0.32, 2.52) | 0.47  (0.20, 1.14) | 0.79  (0.38, 1.63) | **Control** | |

**Note:** The results in the lower left section are network meta-analysis, and the results in the upper right section are traditional paired meta-analysis. Comparisons between treatments should be read from left to right and the estimate is in the cell in common between the upper-left-defining treatment and the lower-right-defining treatment. The relative risks (RR) greater than 1 favour the lower-right-defining treatment. To obtain RRs for comparisons in the opposite direction, reciprocals should be taken. Significant results are in bold and underlined. TIPS: transjugular intrahepatic portal system shunt, EBL: endoscopic band ligation, DOACs: direct oral anticoagulants, LMWH: low molecular weight heparin, NA: not available.

**Supplementary Table 6** Results of traditional paired and network meta-analysis for PVT extension

| **DOACs** | NA | NA | NA | NA | NA | NA | NA | NA | NA | NA | 0.73  (0.18, 3.05) | **0.44**  **(0.32, 0.60)** |
| --- | --- | --- | --- | --- | --- | --- | --- | --- | --- | --- | --- | --- |
| 3.44  (0.65, 18.16) | **LMWH** | NA | NA | NA | NA | NA | NA | NA | NA | NA | **0.21**  **(0.09, 0.50)** |  |
| 10.98  (0.40, 303.96) | 3.19  (0.14, 72.00) | **TIPS** | NA | 0.21  (0.01, 4.12) | NA | NA | NA | NA | 0.09  (0.00, 1.80) | NA | 0.16  (0.01, 3.78) |  |
| 2.05  (0.35, 12.18) | 0.60  (0.15, 2.33) | 0.19  (0.01, 4.50) | **Warfarin** | NA | NA | NA | NA | NA | NA | NA | 0.35  (0.12, 1.03) |  |
| 2.28  (0.03, 198.74) | 0.66  (0.01, 49.71) | 0.21  (0.01, 4.12) | 1.11  (0.01, 87.42) | **EBL *plus* Propranolol** | NA | NA | NA | NA | NA | NA | NA |  |
| 0.99  (0.17, 5.63) | 0.29  (0.08, 1.06) | 0.09  (0.00, 2.12) | 0.48  (0.11, 2.07) | 0.43  (0.01, 33.50) | **LMWH *plus* Warfarin** | NA | NA | NA | NA | NA | 0.74  (0.27, 1.98) |  |
| 24.46  (0.88, 679.88) | 7.10  (0.31, 161.05) | 2.23  (0.04, 111.29) | 11.92  (0.49, 288.25) | 10.71  (0.08, 1468.52) | 24.65  (1.04, 581.70) | **TIPS *plus* Warfarin** | NA | NA | **0.04**  **(0.00, 0.81)** | NA | 0.07  (0.00, 1.71) |  |
| **17.05**  **(1.40, 208.48)** | 4.95  (0.54, 45.86) | 1.55  (0.04, 58.88) | 8.31  (0.82, 84.17) | 7.47  (0.07, 824.66) | 17.19  (1.76, 168.29) | 0.70  (0.02, 26.54) | **LMWH-DOACs sequential** | **0.11**  **(0.02, 0.75)** | NA | NA | NA |  |
| 1.83  (0.38, 8.81) | 0.53  (0.18, 1.56) | 0.17  (0.01, 3.58) | 0.89  (0.25, 3.11) | 0.80  (0.01, 57.94) | 1.84  (0.56, 6.04) | 0.07  (0.00, 1.62) | **0.11**  **(0.02, 0.75)** | **LMWH-Warfarin sequential** | NA | NA | **0.40**  **(0.21, 0.77)** |  |
| 1.49  (0.24, 9.17) | 0.43  (0.11, 1.77) | 0.14  (0.01, 2.50) | 0.73  (0.16, 3.41) | 0.65  (0.01, 42.31) | 1.50  (0.34, 6.70) | 0.06  (0.00, 1.13) | **0.09**  **(0.01, 0.91)** | 0.82  (0.22, 2.99) | **Heparin *plus* DOACs *plus* Warfarin** | NA | 0.43  (0.15, 1.27) |  |
| 1.25  (0.27, 5.82) | 0.36  (0.13, 1.01) | 0.11  (0.01, 2.40) | 0.61  (0.18, 2.04) | 0.55  (0.01, 39.04) | 1.26  (0.40, 3.95) | 0.05  (0.00, 1.08) | **0.07**  **(0.01, 0.62)** | 0.68  (0.28, 1.64) | 0.83  (0.24, 2.95) | **LMWH *plus* DOACs *plus* Warfarin** | 0.59  (0.33, 1.05) |  |
| 0.73  (0.18, 3.05) | **0.21**  **(0.09, 0.50)** | 0.07  (0.00, 1.33) | 0.36  (0.12, 1.03) | 0.32  (0.00, 22.00) | 0.74  (0.27, 1.98) | **0.03**  **(0.00, 0.60)** | **0.04**  **(0.01, 0.33)** | **0.40**  **(0.21, 0.77)** | 0.49  (0.16, 1.50) | 0.59  (0.33, 1.05) | **Control** | |

**Note:** The results in the lower left section are network meta-analysis, and the results in the upper right section are traditional paired meta-analysis. Comparisons between treatments should be read from left to right and the estimate is in the cell in common between the upper-left-defining treatment and the lower-right-defining treatment. The relative risks (RR) greater than 1 favour the lower-right-defining treatment. To obtain RRs for comparisons in the opposite direction, reciprocals should be taken. Significant results are in bold and underlined. TIPS: transjugular intrahepatic portal system shunt, EBL: endoscopic band ligation, DOACs: direct oral anticoagulants, LMWH: low molecular weight heparin, NA: not available.

**Supplementary Table 7** Results of traditional paired and network meta-analysis for hepatic encephalopathy

| **TIPS** | 1.01 (0.62, 1.63) | 0.87 (0.59, 1.26) | **8.23 (2.01, 33.66)** | **3.14 (1.15, 8.54)** | **2.54 (1.39, 4.66)** |
| --- | --- | --- | --- | --- | --- |
| 1.01 (0.62, 1.63) | **EBL *plus* Propranolol** | NA | NA | NA |  |
| 0.87 (0.59, 1.26) | 0.86 (0.47, 1.59) | **TIPS *plus* Warfarin** | **9.43 (2.37, 37.51)** | **3.59 (1.37, 9.41)** |  |
| **8.19 (2.01, 33.32)** | **8.14 (1.84, 35.91)** | **9.45 (2.38, 37.56)** | **Heparin *plus* DOACs *plus* Warfarin** | 0.38 (0.07, 1.99) |  |
| **3.12 (1.15, 8.44)** | **3.10 (1.03, 9.37)** | **3.60 (1.38, 9.42)** | 0.38 (0.07, 1.99) | **Control** | |

**Note:** The results in the lower left section are network meta-analysis, and the results in the upper right section are traditional paired meta-analysis. Comparisons between treatments should be read from left to right and the estimate is in the cell in common between the upper-left-defining treatment and the lower-right-defining treatment. The relative risks (RR) greater than 1 favour the lower-right-defining treatment. To obtain RRs for comparisons in the opposite direction, reciprocals should be taken. Significant results are in bold and underlined. TIPS: transjugular intrahepatic portal system shunt, EBL: endoscopic band ligation, DOACs: direct oral anticoagulants, LMWH: low molecular weight heparin, NA: not available.

**Supplementary Table 8** Test of inconsistency for complete recanalization

| **Side** | **Direct** | |  | **Indirect** | |  | **Difference** | | | **tau** |
| --- | --- | --- | --- | --- | --- | --- | --- | --- | --- | --- |
|  | **Coef.** | **Std. Err.** |  | **Coef.** | **Std. Err.** |  | **Coef.** | **Std. Err.** | **P>z** |  |
| 01 02 | -0.3806658 | 0.2883073 |  | -0.6133592 | 0.6133284 |  | 0.2326933 | 0.6702016 | 0.728 | 3.02E-06 |
| 01 04 | 0.0294455 | 0.2693523 |  | -0.5519272 | 1.138475 |  | 0.5813727 | 1.160452 | 0.616 | 3.53E-08 |
| 01 10* | -0.7802192 | 0.2531676 |  | -0.6582498 | 0.6194028 |  | -0.1219694 | 0.6351616 | 0.848 | 1.50E-07 |
| 02 04 | 0.4177564 | 0.2582694 |  | 0.4473763 | 0.515294 |  | -0.0296198 | 0.5857375 | 0.96 | 3.62E-07 |
| 02 10* | -0.341739 | 0.1744639 |  | -0.6398301 | 1.170735 |  | 0.2980911 | 1.176351 | 0.8 | 3.00E-06 |
| 03 05* | -0.7081998 | 0.1956435 |  | -2.646315 | 141.5565 |  | 1.938115 | 141.5564 | 0.989 | 9.72E-08 |
| 03 06* | 0.0683055 | 0.0376726 |  | 2.091373 | 170.0143 |  | -2.023067 | 170.0143 | 0.991 | 0.0000969 |
| 03 09* | -1.313999 | 0.2460387 |  | -1.249711 | 0.6318562 |  | -0.0642882 | 0.6232865 | 0.918 | 0.1056869 |
| 03 10* | -1.764264 | 0.4045995 |  | -1.493034 | 0.6147227 |  | -0.2712302 | 0.5398576 | 0.615 | 0.0770888 |
| 04 10* | -0.7655295 | 0.209067 |  | -0.825537 | 0.6580232 |  | 0.0600075 | 0.681989 | 0.93 | 1.51E-07 |
| 06 09* | -1.371069 | 0.2325591 |  | -1.642249 | 0.5613082 |  | 0.2711797 | 0.539303 | 0.615 | 0.0770503 |
| 06 10* | -1.804107 | 0.4111613 |  | -1.868278 | 0.6731056 |  | 0.064171 | 0.6233359 | 0.918 | 0.1057424 |
| 07 08* | -1.252763 | 0.3933979 |  | 1.351485 | 199.9799 |  | -2.604247 | 199.9811 | 0.99 | 9.55E-09 |
| 08 10* | -0.8168998 | 0.341422 |  | 0.0841229 | 81.62945 |  | -0.9010227 | 81.63064 | 0.991 | 3.42E-07 |
| 09 10* | -0.4267852 | 0.4325416 |  | 0.9999078 | 89.43411 |  | -1.426693 | 89.43652 | 0.987 | 0.0000399 |

**Note:** 1: DOACs, 2: LMWH, 3: TIPS, 4: Warfarin, 5: EBL *plus* Propranolol, 6: TIPS *plus* Warfarin, 7: LMWH-DOACss sequential, 8: LMWH-Warfarin sequential, 9: Heparin *plus* DOACs *plus* Warfarin, 10: Control. * Warning: all the evidence about these contrasts comes from the trials which directly compare them.

**Supplementary Table 9** Test of inconsistency for partial recanalization

| **Side** | **Direct** | |  | **Indirect** | |  | **Difference** | | | **tau** |
| --- | --- | --- | --- | --- | --- | --- | --- | --- | --- | --- |
|  | **Coef.** | **Std. Err.** |  | **Coef.** | **Std. Err.** |  | **Coef.** | **Std. Err.** | **P>z** |  |
| 1 9 * | -1.919935 | 1.044462 |  | -1.094377 | 33.34849 |  | -0.8255574 | 33.36484 | 0.98 | 5.62E-08 |
| 2 4 * | 0.6254806 | 0.4304175 |  | 1.760603 | 141.727 |  | -1.135122 | 141.7263 | 0.994 | 1.35E-09 |
| 2 5 * | -0.6531496 | 0.3446329 |  | -1.788998 | 141.4195 |  | 1.135849 | 141.4199 | 0.994 | 2.06E-07 |
| 2 8 * | 0.2964156 | 0.4412579 |  | 1.005713 | 1.193952 |  | -0.709297 | 1.269445 | 0.576 | 1.40E-07 |
| 2 9 * | -2.021974 | 0.9087216 |  | -0.7214165 | 0.8810748 |  | -1.300558 | 1.269591 | 0.306 | 6.57E-10 |
| 3 9 * | -0.9162907 | 0.74642 |  | -2.922178 | 200.0209 |  | 2.005887 | 200.0251 | 0.992 | 1.00E-09 |
| 5 8 * | 1.211814 | 0.4617843 |  | -0.0889897 | 1.175907 |  | 1.300803 | 1.269554 | 0.306 | 8.70E-10 |
| 5 9 * | -0.3192199 | 0.9320839 |  | -1.029065 | 0.8705189 |  | 0.7098455 | 1.26965 | 0.576 | 5.22E-09 |
| 6 7 * | -0.6931472 | 0.7071068 |  | -2.410761 | 199.9956 |  | 1.717614 | 199.9993 | 0.993 | 1.12E-08 |
| 7 9 * | -0.3672529 | 0.2595772 |  | -1.97481 | 81.70515 |  | 1.607558 | 81.70584 | 0.984 | 1.20E-08 |
| 8 9 * | -1.731867 | 0.4997375 |  | -2.715233 | 89.45856 |  | 0.9833668 | 89.46253 | 0.991 | 2.03E-07 |

**Note:** 1: DOACs, 2: TIPS, 3: Warfarin, 4: EBL *plus* Propranolol, 5: TIPS *plus* Warfarin, 6: LMWH-DOACss sequential, 7: LMWH-Warfarin sequential, 8: Heparin *plus* DOACs *plus* Warfarin, 9: Control. * Warning: all the evidence about these contrasts comes from the trials which directly compare them.

**Supplementary Table 10** Test of inconsistency for bleeding

| **Side** | **Direct** | |  | **Indirect** | |  | **Difference** | | | **tau** |
| --- | --- | --- | --- | --- | --- | --- | --- | --- | --- | --- |
|  | **Coef.** | **Std. Err.** |  | **Coef.** | **Std. Err.** |  | **Coef.** | **Std. Err.** | **P>z** |  |
| 01 10* | -1.098612 | 1.220011 |  | 0.1712165 | 30.15137 |  | -1.269829 | 30.17605 | 0.966 | 0.4528717 |
| 02 10* | 0.6751287 | 0.7596637 |  | -2.868724 | 200.0125 |  | 3.543852 | 200.0158 | 0.986 | 0.4527814 |
| 03 04* | 1.05779 | 0.6245164 |  | 4.786617 | 200.0473 |  | -3.728826 | 200.0473 | 0.985 | 0.4527922 |
| 03 06* | 0.8486143 | 0.4802871 |  | -2.880308 | 199.9258 |  | 3.728922 | 199.9265 | 0.985 | 0.4527897 |
| 03 09* | 0.9084788 | 0.7252752 |  | 0.5785125 | 1.475015 |  | 0.3299663 | 1.644 | 0.841 | 0.6400106 |
| 03 10* | 0.4068989 | 0.456474 |  | 2.191433 | 0.9747423 |  | -1.784534 | 1.07847 | 0.098 | 0.1965683 |
| 05 10* | -0.0748322 | 0.4172866 |  | -2.119161 | 115.4402 |  | 2.044329 | 115.4418 | 0.986 | 0.4528056 |
| 06 09* | 0.2531472 | 0.3028883 |  | -1.532194 | 1.030826 |  | 1.785341 | 1.078548 | 0.098 | 0.1965867 |
| 06 10* | -0.1716815 | 0.7189498 |  | 0.1569876 | 1.479565 |  | -0.3286691 | 1.644113 | 0.842 | 0.6400243 |
| 07 08* | -0.8109302 | 0.9772488 |  | -1.318189 | 200.0354 |  | 0.5072583 | 200.0415 | 0.998 | 0.4527859 |
| 08 10* | -0.0319714 | 0.5977568 |  | -0.9111736 | 81.66759 |  | 0.8792023 | 81.67084 | 0.991 | 0.4527956 |
| 09 10* | -0.0842893 | 0.4504959 |  | -3.44912 | 100.0252 |  | 3.36483 | 100.0267 | 0.973 | 0.452837 |

**Note:** 1: DOACs, 2: LMWH, 3: TIPS, 4: EBL *plus* Propranolol, 5: LMWH *plus* Warfarin, 6: TIPS *plus* Warfarin, 7: LMWH-DOACs sequential, 8: LMWH-Warfarin sequential, 9: Heparin *plus* DOACs *plus* Warfarin, 10: Control. * Warning: all the evidence about these contrasts comes from the trials which directly compare them.

**Supplementary Table 11** Test of inconsistency for major bleeding

| **Side** | **Direct** | |  | **Indirect** | |  | **Difference** | | | **tau** |
| --- | --- | --- | --- | --- | --- | --- | --- | --- | --- | --- |
|  | **Coef.** | **Std. Err.** |  | **Coef.** | **Std. Err.** |  | **Coef.** | **Std. Err.** | **P>z** |  |
| 1 2 * | 1.232144 | 1.022563 |  | 1.277348 | 3.226799 |  | -0.0452039 | 3.625518 | 0.99 | 7.84E-09 |
| 1 4 * | 0.9830675 | 0.8669987 |  | -1.981656 | 3.192247 |  | 2.964724 | 3.160925 | 0.348 | 1.42E-09 |
| 1 8 * | 0.9058492 | 0.9221292 |  | 2.493394 | 2.099561 |  | -1.587544 | 2.300534 | 0.49 | 5.90E-08 |
| 2 4 * | -0.2135741 | 0.5592217 |  | -2.009796 | 2.365428 |  | 1.796222 | 2.475616 | 0.468 | 3.32E-07 |
| 2 8 * | -0.0949166 | 0.3725198 |  | 2.870934 | 3.160776 |  | -2.965851 | 3.162602 | 0.348 | 3.82E-06 |
| 3 7 * | 0.5753702 | 0.36756 |  | -0.7294409 | 2.366589 |  | 1.304811 | 2.384925 | 0.584 | 9.62E-08 |
| 3 8 * | 0.405466 | 0.4120954 |  | 1.71032 | 2.344318 |  | -1.304854 | 2.384776 | 0.584 | 7.92E-09 |
| 4 8 * | 0.2663117 | 0.4737506 |  | 0.2190902 | 3.548767 |  | 0.0472215 | 3.623643 | 0.99 | 2.38E-06 |
| 5 8 * | 2.032922 | 1.492526 |  | 0.2989758 | 200.0066 |  | 1.733946 | 200.0233 | 0.993 | 2.07E-11 |
| 6 7 * | 0.377929 | 0.2895043 |  | -0.9268823 | 2.355728 |  | 1.304811 | 2.384924 | 0.584 | 3.03E-06 |
| 6 8 * | 0.2080299 | 0.3442891 |  | 1.512883 | 2.333364 |  | -1.304853 | 2.384777 | 0.584 | 1.47E-06 |
| 7 8 * | -0.1030045 | 0.3617715 |  | 1.885289 | 141.3932 |  | -1.988294 | 141.3942 | 0.989 | 4.99E-08 |

**Note:** 1: DOACs, 2: LMWH, 3: TIPS, 4: Warfarin, 5: LMWH *plus* Warfarin, 6: TIPS *plus* Warfarin, 7: Heparin *plus* DOACs *plus* Warfarin, 8: Control. * Warning: all the evidence about these contrasts comes from the trials which directly compare them.

**Supplementary Table 12** Test of inconsistency for mortality

| **Side** | **Direct** | |  | **Indirect** | |  | **Difference** | | | **tau** |
| --- | --- | --- | --- | --- | --- | --- | --- | --- | --- | --- |
|  | **Coef.** | **Std. Err.** |  | **Coef.** | **Std. Err.** |  | **Coef.** | **Std. Err.** | **P>z** |  |
| 1 2 | 0.1720373 | 0.7085394 |  | -0.0495807 | 1.343417 |  | 0.221618 | 1.526792 | 0.885 | 0.3686178 |
| 1 4 * | -0.0878502 | 0.5273241 |  | -3.106547 | 3.075372 |  | 3.018697 | 3.10995 | 0.332 | 0.2869664 |
| 1 8 | 0.4955435 | 0.6212966 |  | 1.424819 | 1.18622 |  | -0.9292752 | 1.327293 | 0.484 | 0.3464203 |
| 2 4 | -0.2561917 | 0.6668985 |  | -0.4161728 | 1.255435 |  | 0.159981 | 1.434744 | 0.911 | 0.3705533 |
| 2 8 * | 0.5241645 | 0.4070267 |  | 3.54524 | 3.106686 |  | -3.021076 | 3.112161 | 0.332 | 0.2869724 |
| 3 5 * | 0.0276511 | 0.382857 |  | -1.055414 | 141.2226 |  | 1.083065 | 141.222 | 0.994 | 0.293259 |
| 3 6 * | -0.6218515 | 0.4871693 |  | -0.4585881 | 2.043124 |  | -0.1632634 | 2.107627 | 0.938 | 0.3356548 |
| 3 7 * | -0.0991833 | 0.36059 |  | 0.9838644 | 141.4471 |  | -1.083048 | 141.4479 | 0.994 | 0.2932589 |
| 3 8 * | 0.2012788 | 0.4084757 |  | -1.524664 | 2.045234 |  | 1.725943 | 2.086905 | 0.408 | 0.3080761 |
| 4 8 * | 0.8387133 | 0.5691564 |  | 1.059765 | 1.412172 |  | -0.2210516 | 1.526726 | 0.885 | 0.3686218 |
| 6 7 * | 0.5988305 | 0.4508906 |  | -1.130189 | 2.029135 |  | 1.72902 | 2.088295 | 0.408 | 0.3081313 |
| 6 8 * | 0.749272 | 0.4470464 |  | 1.595226 | 89.4402 |  | -0.8459536 | 89.44158 | 0.992 | 0.2932809 |
| 7 8 * | 0.2424135 | 0.4107455 |  | 0.0800392 | 2.06418 |  | 0.1623743 | 2.107573 | 0.939 | 0.335657 |

**Note:** 1: DOACs, 2: LMWH, 3: TIPS, 4: Warfarin, 5: EBL *plus* Propranolol, 6: TIPS *plus* Warfarin, 7: Heparin *plus* DOACs *plus* Warfarin, 8: Control. * Warning: all the evidence about these contrasts comes from the trials which directly compare them.

**Supplementary Table 13** Test of inconsistency for PVT extension

| **Side** | **Direct** | |  | **Indirect** | |  | **Difference** | | | **tau** |
| --- | --- | --- | --- | --- | --- | --- | --- | --- | --- | --- |
|  | **Coef.** | **Std. Err.** |  | **Coef.** | **Std. Err.** |  | **Coef.** | **Std. Err.** | **P>z** |  |
| 01 12* | 0.3136576 | 0.7289558 |  | 1.416417 | 30.15066 |  | -1.10276 | 30.15947 | 0.971 | 9.00E-09 |
| 02 12* | 1.550597 | 0.4344475 |  | -0.9221122 | 200.0259 |  | 2.47271 | 200.0273 | 0.99 | 1.08E-07 |
| 03 05* | 1.570217 | 1.52366 |  | 6.364253 | 200.0675 |  | -4.794036 | 200.0617 | 0.981 | 2.41E-09 |
| 03 07* | -0.8001074 | 1.995825 |  | -5.59417 | 200.0056 |  | 4.794063 | 200.0155 | 0.981 | 3.01E-08 |
| 03 10* | 2.356768 | 1.502278 |  | -1.45546 | 2.619711 |  | 3.812228 | 2.383912 | 0.11 | 3.64E-09 |
| 03 12* | 1.848176 | 1.62205 |  | 5.657474 | 2.395943 |  | -3.809298 | 2.383847 | 0.11 | 1.45E-09 |
| 04 12* | 1.033157 | 0.5430674 |  | -0.4047217 | 141.421 |  | 1.437878 | 141.4241 | 0.992 | 5.36E-08 |
| 06 12* | 0.3063742 | 0.504262 |  | 0.3222094 | 199.8984 |  | -0.0158352 | 199.9003 | 1 | 2.95E-07 |
| 07 10* | 3.15664 | 1.504565 |  | -0.6559382 | 2.621684 |  | 3.812578 | 2.384234 | 0.11 | 6.19E-09 |
| 07 12* | 2.64921 | 1.624133 |  | 6.460322 | 2.398082 |  | -3.811112 | 2.384202 | 0.11 | 1.08E-08 |
| 08 09* | 2.233592 | 0.9940298 |  | -3.440215 | 199.9807 |  | 5.673808 | 199.9881 | 0.977 | 3.23E-09 |
| 09 12* | 0.9174828 | 0.3354591 |  | -1.376115 | 81.64416 |  | 2.293598 | 81.64531 | 0.978 | 2.49E-07 |
| 10 12* | 0.7152274 | 0.5717404 |  | -2.748818 | 115.493 |  | 3.464045 | 115.4962 | 0.976 | 4.06E-07 |
| 11 12* | 0.5338752 | 0.2962865 |  | 0.0947037 | 200.2836 |  | 0.4391715 | 200.2842 | 0.998 | 7.70E-09 |

**Note:** 1: DOACs, 2: LMWH, 3: TIPS, 4: Warfarin, 5: EBL *plus* Propranolol, 6: LMWH *plus* Warfarin, 7: TIPS *plus* Warfarin, 8: LMWH-DOACs sequential, 9: LMWH-Warfarin sequential, 10: Heparin *plus* DOACs *plus* Warfarin, 11: LMWH *plus* DOACs *plus* Warfarin, 12: Control. * Warning: all the evidence about these contrasts comes from the trials which directly compare them.

**Supplementary Table 14** Test of inconsistency for hepatic encephalopathy

| **Side** | **Direct** | |  | **Indirect** | |  | **Difference** | | | **tau** |
| --- | --- | --- | --- | --- | --- | --- | --- | --- | --- | --- |
|  | **Coef.** | **Std. Err.** |  | **Coef.** | **Std. Err.** |  | **Coef.** | **Std. Err.** | **P>z** |  |
| 1 3 * | -0.9650809 | 0.8445671 |  | -3.232417 | 115.5087 |  | 2.267336 | 115.5096 | 0.984 | 4.00E-09 |
| 1 4 * | 1.143003 | 0.5111454 |  | 1.043215 | 1.136809 |  | 0.0997878 | 1.076872 | 0.926 | 1.50E-08 |
| 1 5 * | 1.279163 | 0.4909435 |  | 1.378379 | 1.16333 |  | -0.0992164 | 1.076981 | 0.927 | 3.81E-08 |
| 2 4 * | 0.0058327 | 0.2466856 |  | 2.268652 | 141.5715 |  | -2.262819 | 141.5722 | 0.987 | 1.44E-07 |
| 3 4 * | 2.108145 | 0.7184996 |  | 2.00853 | 1.244073 |  | 0.0996147 | 1.077002 | 0.926 | 8.99E-08 |
| 3 5 * | 2.244321 | 0.7042727 |  | 2.343745 | 1.268275 |  | -0.0994242 | 1.077039 | 0.926 | 4.78E-08 |
| 4 5 * | 0.1437214 | 0.1931723 |  | 2.409923 | 141.2818 |  | -2.266202 | 141.282 | 0.987 | 1.23E-07 |

**Note:** 1: TIPS, 2: EBL *plus* Propranolol, 3: TIPS *plus* Warfarin, 4: Heparin *plus* DOACs *plus* Warfarin, 5: Control. * Warning: all the evidence about these contrasts comes from the trials which directly compare them.

**Supplemental Table 15** Results of sensitivity analyses for complete recanalization

| **Anticoagulant Drugs** | **Main results** | | |  | **Results of sensitivity analysis** | | |
| --- | --- | --- | --- | --- | --- | --- | --- |
|  | **RR, 95%CI** | **SUCRA** | **Rank** |  | **RR, 95%CI** | **SUCRA** | **Rank** |
| LMWH-DOACs sequential | 7.92 (2.85, 21.99) | 92.7 | 1 |  | 7.00 (2.30, 21.30) | 87.2 | 2 |
| TIPS *plus* Warfarin | 6.08 (2.83, 13.08) | 91.3 | 2 |  | 6.08 (2.83, 13.08) | 91.2 | 1 |
| TIPS | 5.68 (2.63, 12.24) | 80.3 | 3 |  | 5.68 (2.63, 12.24) | 78.8 | 3 |
| EBL *plus* Propranolol | 2.80 (1.18, 6.60) | 55.0 | 4 |  | 3.44 (1.41, 8.42) | 59.0 | 4 |
| LMWH-Warfarin sequential | 2.26 (1.16, 4.42) | 47.0 | 5 |  | 2.00 (0.90, 4.46) | 40.7 | 5 |
| Warfarin | 2.16 (1.46, 3.21) | 44.9 | 6 |  | 2.00 (0.62, 6.45) | 39.7 | 6 |
| DOAC | 2.15 (1.33, 3.48) | 44.5 | 7 |  | NA | NA | NA |
| Heparin *plus* DOACs *plus* Warfarin | 1.53 (0.66, 3.58) | 23.3 | 8 |  | 1.53 (0.66, 3.58) | 28.2 | 7 |
| LMWH | 1.41 (1.01, 1.99) | 18.7 | 9 |  | 1.17 (0.64, 2.14) | 16.8 | 8 |
| Control | 1 | 2.4 | 10 |  | 1 | 8.3 | 9 |

**Note:** The results of RRs are network comparisons of anticoagulant drugs and control. CIs: confidence intervals, DOACs: direct oral anticoagulants, EBL: endoscopic band ligation, LMWH: low molecular weight heparin, RR: relative risks, NA: not available, SUCRA: surface under the cumulative ranking curve, TIPS: transjugular intrahepatic portal system shunt.

**Supplemental Table 16** Results of sensitivity analyses for partial recanalization

| **Anticoagulant Drugs** | **Main results** | | |  | **Results of sensitivity analysis** | | |
| --- | --- | --- | --- | --- | --- | --- | --- |
|  | **RR, 95%CI** | **SUCRA** | **Rank** |  | **RR, 95%CI** | **SUCRA** | **Rank** |
| EBL *plus* Propranolol | 7.22 (1.62, 32.25) | 84.2 | 1 |  | 7.42 (0.98, 56.12) | 82.7 | 1 |
| Heparin *plus* DOACs *plus* Warfarin | 5.65 (2.12, 15.05) | 77.3 | 2 |  | 5.65 (2.12, 15.05) | 81.6 | 2 |
| DOAC | 6.81 (0.88, 52.73) | 75.2 | 3 |  | NA | NA | NA |
| TIPS | 3.86 (1.12, 13.30) | 58.7 | 4 |  | 3.86 (1.12, 13.30) | 64.6 | 3 |
| LMWH-DOACs sequential | 2.89 (0.66, 12.64) | 49.6 | 5 |  | 3.00 (0.64, 14.04) | 55.2 | 4 |
| Warfarin | 2.50 (0.58, 10.80) | 43.8 | 6 |  | 2.50 (0.58, 10.80) | 47.6 | 5 |
| TIPS *plus* Warfarin | 2.01 (0.57, 7.04) | 32.2 | 7 |  | 2.01 (0.57, 7.04) | 33.8 | 6 |
| LMWH-Warfarin sequential | 1.44 (0.87, 2.40) | 22.8 | 8 |  | 1.50 (0.76, 2.96) | 26.7 | 7 |
| Control | 1 | 6.2 | 9 |  | 1 | 7.7 | 8 |

**Note:** The results of RRs are network comparisons of anticoagulant drugs and control. CIs: confidence intervals, DOACs: direct oral anticoagulants, EBL: endoscopic band ligation, LMWH: low molecular weight heparin, RR: relative risks, NA: not available, SUCRA: surface under the cumulative ranking curve, TIPS: transjugular intrahepatic portal system shunt.

**Supplemental Table 17** Results of sensitivity analyses for bleeding

| **Anticoagulant Drugs** | **Main results** | | |  | **Results of sensitivity analysis** | | |
| --- | --- | --- | --- | --- | --- | --- | --- |
|  | **RR, 95%CI** | **SUCRA** | **Rank** |  | **RR, 95%CI** | **SUCRA** | **Rank** |
| TIPS | 0.46 (0.16, 1.36) | 87.5 | 1 |  | 0.46 (0.15, 1.40) | 89.9 | 1 |
| LMWH | 0.51 (0.11, 2.26) | 77.5 | 2 |  | NA | NA | NA |
| Control | 1 | 52.0 | 3 |  | 1 | 51.8 | 3 |
| LMWH-Warfarin sequential | 1.03 (0.32, 3.33) | 50.1 | 4 |  | 0.89 (0.25, 3.17) | 57.1 | 2 |
| LMWH *plus* Warfarin | 1.08 (0.48, 2.44) | 48.8 | 5 |  | 1.07 (0.46, 2.51) | 47.8 | 4 |
| TIPS *plus* Warfarin | 1.08 (0.40, 2.93) | 48.8 | 6 |  | 1.09 (0.39, 3.06) | 43.5 | 6 |
| LMWH *plus* DOACs *plus* Warfarin | 1.09 (0.45, 2.63) | 48.0 | 7 |  | NA | NA | NA |
| EBL *plus* Propranolol | 1.34 (0.26, 6.82) | 39.7 | 8 |  | 1.33 (0.25, 7.17) | 38.5 | 7 |
| LMWH-DOACs sequential | 2.32 (0.25, 21.93) | 25.8 | 9 |  | 2.00 (0.20, 20.35) | 27.0 | 8 |
| DOAC | 2.99 (0.27, 32.64) | 22.0 | 10 |  | NA | NA | NA |
| Heparin *plus* DOACs *plus* Warfarin | NA | NA | 11 |  | 1.08 (0.44, 2.69) | 44.4 | 5 |

**Note:** The results of RRs are network comparisons of anticoagulant drugs and control. CIs: confidence intervals, DOACs: direct oral anticoagulants, EBL: endoscopic band ligation, LMWH: low molecular weight heparin, RR: relative risks, NA: not available, SUCRA: surface under the cumulative ranking curve, TIPS: transjugular intrahepatic portal system shunt.

**Supplemental Table 18** Results of sensitivity analyses for major bleeding

| **Anticoagulant Drugs** | **Main results** | | |  | **Results of sensitivity analysis** | | |
| --- | --- | --- | --- | --- | --- | --- | --- |
|  | **RR, 95%CI** | **SUCRA** | **Rank** |  | **RR, 95%CI** | **SUCRA** | **Rank** |
| LMWH *plus* Warfarin | 0.13 (0.01, 2.44) | 85.4 | 1 |  | 0.13 (0.01, 2.44) | 76.5 | 2 |
| DOAC | 0.31 (0.06, 1.62) | 77.9 | 2 |  | 0.08 (0.00, 4.92) | 79.2 | 1 |
| TIPS | 0.64 (0.29, 1.41) | 63.1 | 3 |  | 0.64 (0.29, 1.41) | 51.3 | 4 |
| TIPS *plus* Warfarin | 0.78 (0.40, 1.51) | 50.4 | 4 |  | 0.78 (0.40, 1.51) | 40.1 | 5 |
| Warfarin | 0.77 (0.31, 1.90) | 47.6 | 5 |  | 0.20 (0.01, 3.82) | 65.7 | 3 |
| Control | 1 | 28.5 | 6 |  | 1 | 22.4 | 6 |
| LMWH | 1.08 (0.52, 2.23) | 24.0 | 7 |  | NA | NA | NA |
| Heparin *plus* DOACs *plus* Warfarin | 1.11 (0.55, 2.25) | 23.1 | 8 |  | 1.11 (0.55, 2.25) | 14.9 | 7 |

**Note:** The results of RRs are network comparisons of anticoagulant drugs and control. CIs: confidence intervals, DOACs: direct oral anticoagulants, EBL: endoscopic band ligation, LMWH: low molecular weight heparin, RR: relative risks, NA: not available, SUCRA: surface under the cumulative ranking curve, TIPS: transjugular intrahepatic portal system shunt.

**Supplemental Table 1 9** Results of sensitivity analyses for mortality

| **Anticoagulant Drugs** | **Main results** | | |  | **Results of sensitivity analysis** | | |
| --- | --- | --- | --- | --- | --- | --- | --- |
|  | **RR, 95%CI** | **SUCRA** | **Rank** |  | **RR, 95%CI** | **SUCRA** | **Rank** |
| Warfarin | 0.43 (0.16,1.13) | 77.8 | 1 |  | 0.11 (0.01,1.89) | 81.8 | 1 |
| Heparin *plus* DOACs *plus* Warfarin | 0.47 (0.20,1.14) | 74.1 | 2 |  | 0.46 (0.23,0.92) | 61.8 | 4 |
| DOAC | 0.51 (0.18,1.46) | 67.0 | 3 |  | 0.12 (0.01,2.79) | 77.2 | 2 |
| LMWH | 0.57 (0.25,1.30) | 60.9 | 4 |  | NA | NA | NA |
| TIPS *plus* Warfarin | 0.79 (0.38,1.63) | 38.7 | 5 |  | 0.79 (0.50,1.25) | 31.2 | 5 |
| TIPS | 0.87 (0.40,1.90) | 31.5 | 6 |  | 0.84 (0.50,1.42) | 24.4 | 6 |
| EBL *plus* Propranolol | 0.90 (0.32,2.52) | 30.3 | 7 |  | 0.41 (0.12,1.32) | 62.4 | 3 |
| Control | 1 | 19.7 | 8 |  | 1 | 11.4 | 7 |

**Note:** The results of RRs are network comparisons of anticoagulant drugs and control. CIs: confidence intervals, DOACs: direct oral anticoagulants, EBL: endoscopic band ligation, LMWH: low molecular weight heparin, RR: relative risks, SUCRA: surface under the cumulative ranking curve, TIPS: transjugular intrahepatic portal system shunt.

**Supplemental Table 20** Results of sensitivity analyses for PVT extension

| **Anticoagulant Drugs** | **Main results** | | |  | **Results of sensitivity analysis** | | |
| --- | --- | --- | --- | --- | --- | --- | --- |
|  | **RR, 95%CI** | **SUCRA** | **Rank** |  | **RR, 95%CI** | **SUCRA** | **Rank** |
| TIPS *plus* Warfarin | 0.03 (0.00, 0.60) | 88.8 | 1 |  | 0.03 (0.00, 0.80) | 84.1 | 1 |
| LMWH-DOACs sequential | 0.04 (0.01, 0.33) | 87.5 | 2 |  | 0.04 (0.00, 0.57) | 84.0 | 2 |
| TIPS | 0.07 (0.00, 1.33) | 79.0 | 3 |  | 0.07 (0.00, 1.77) | 74.1 | 3 |
| LMWH | 0.21 (0.09, 0.50) | 67.2 | 4 |  | NA | NA | NA |
| Warfarin | 0.36 (0.12, 1.03) | 51.2 | 5 |  | 0.35 (0.10, 1.29) | 47.5 | 4 |
| LMWH-Warfarin sequential | 0.40 (0.21, 0.77) | 47.0 | 6 |  | 0.42 (0.11, 1.60) | 43.7 | 5 |
| EBL *plus* Propranolol | 0.32 (0.00, 22.00) | 46.9 | 7 |  | 0.35 (0.00, 30.76) | 41.7 | 6 |
| Heparin *plus* DOACs *plus* Warfarin | 0.49 (0.16, 1.50) | 40.0 | 8 |  | 0.52 (0.13, 2.07) | 34.1 | 7 |
| LMWH *plus* DOACs *plus* Warfarin | 0.59 (0.33, 1.05) | 32.1 | 9 |  | NA | NA | NA |
| DOAC | 0.73 (0.18, 3.05) | 25.9 | 10 |  | NA | NA | NA |
| LMWH *plus* Warfarin | 0.74 (0.27, 1.98) | 24.0 | 11 |  | 0.74 (0.19, 2.90) | 26.6 | 8 |
| Control | 1 | 10.5 | 12 |  | 1 | 14.3 | 9 |

**Note:** The results of RRs are network comparisons of anticoagulant drugs and control. CIs: confidence intervals, DOACs: direct oral anticoagulants, EBL: endoscopic band ligation, LMWH: low molecular weight heparin, RR: relative risks, NA: not available, SUCRA: surface under the cumulative ranking curve, TIPS: transjugular intrahepatic portal system shunt.

**Supplemental Table 21** Results of sensitivity analyses for hepatic encephalopathy

| **Anticoagulant Drugs** | **Main results** | | |  | **Results of sensitivity analysis** | | |
| --- | --- | --- | --- | --- | --- | --- | --- |
|  | **RR, 95%CI** | **SUCRA** | **Rank** |  | **RR, 95%CI** | **SUCRA** | **Rank** |
| LMWH *plus* DOACs *plus* Warfarin | 0.38 (0.07, 1.99) | 96.4 | 1 |  | 0.38 (0.07, 1.99) | 95.6 | 1 |
| Control | 1 | 77.3 | 2 |  | 1 | 73.4 | 2 |
| TIPS | 3.12 (1.15, 8.44) | 33.1 | 3 |  | 3.12 (1.15, 8.44) | 25.2 | 4 |
| EBL *plus* Propranolol | 3.10 (1.03, 9.37) | 29.0 | 4 |  | 2.00 (0.44, 9.03) | 46.0 | 3 |
| TIPS *plus* Warfarin | 3.60 (1.38, 9.42) | 14.1 | 5 |  | 3.60 (1.38, 9.42) | 9.8 | 5 |

**Note:** The results of RRs are network comparisons of anticoagulant drugs and control. CIs: confidence intervals, DOACs: direct oral anticoagulants, EBL: endoscopic band ligation, LMWH: low molecular weight heparin, RR: relative risks, SUCRA: surface under the cumulative ranking curve, TIPS: transjugular intrahepatic portal system shunt.

**Supplementary Figure 1** Network plot for bleeding


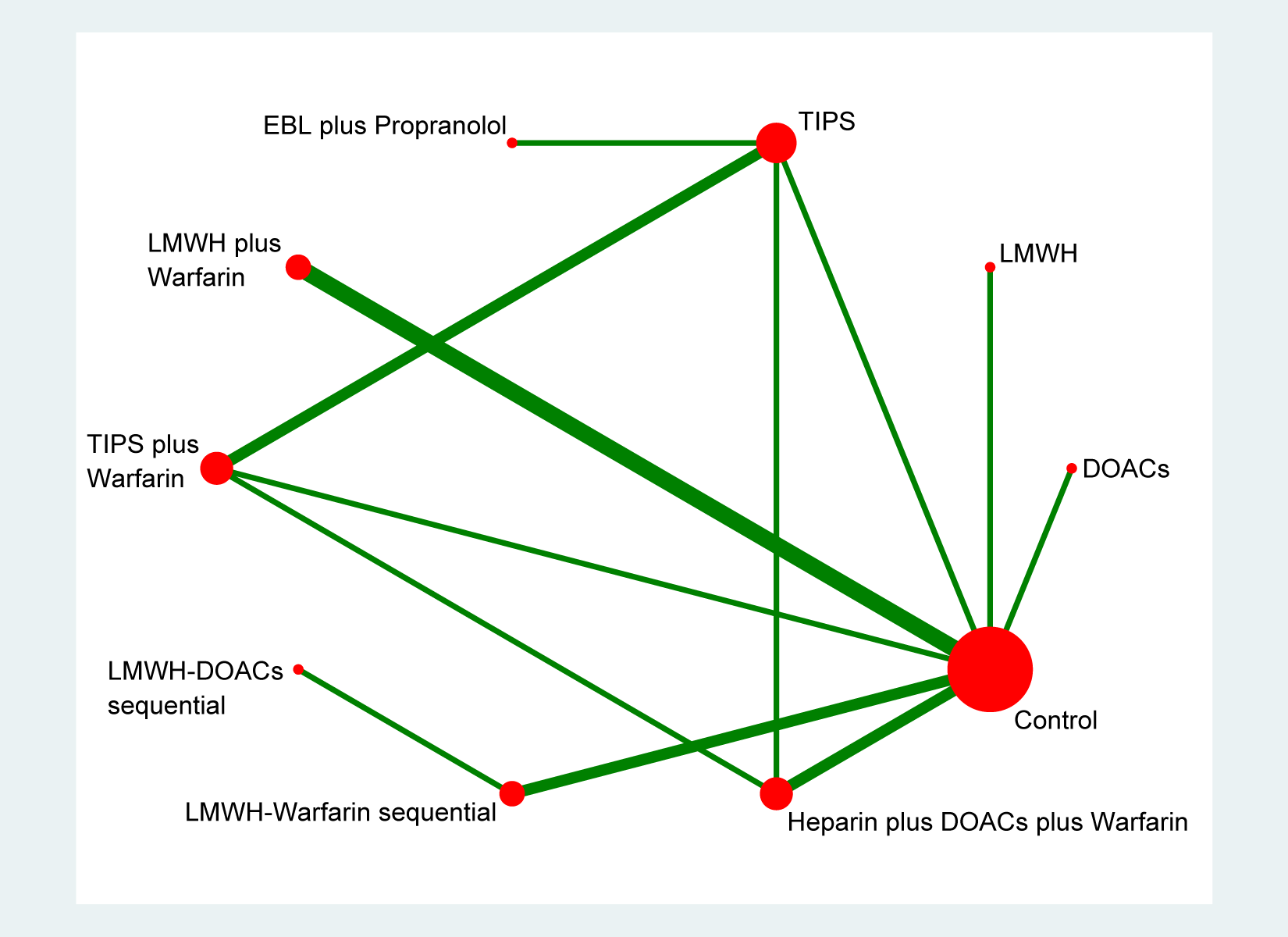


**Supplementary Figure 2** Network plot for major bleeding


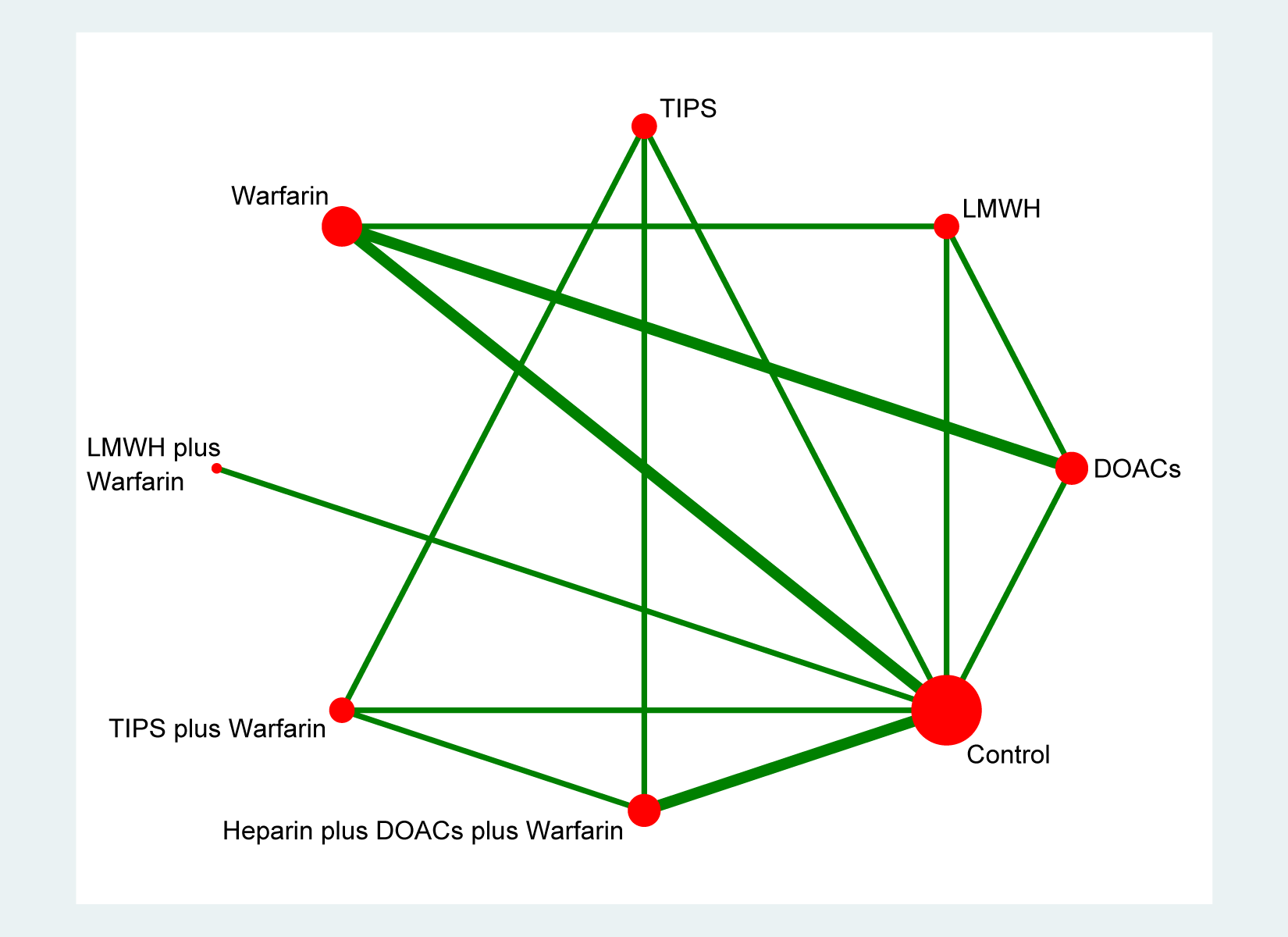


**Supplementary Figure 3** Network plot for mortality


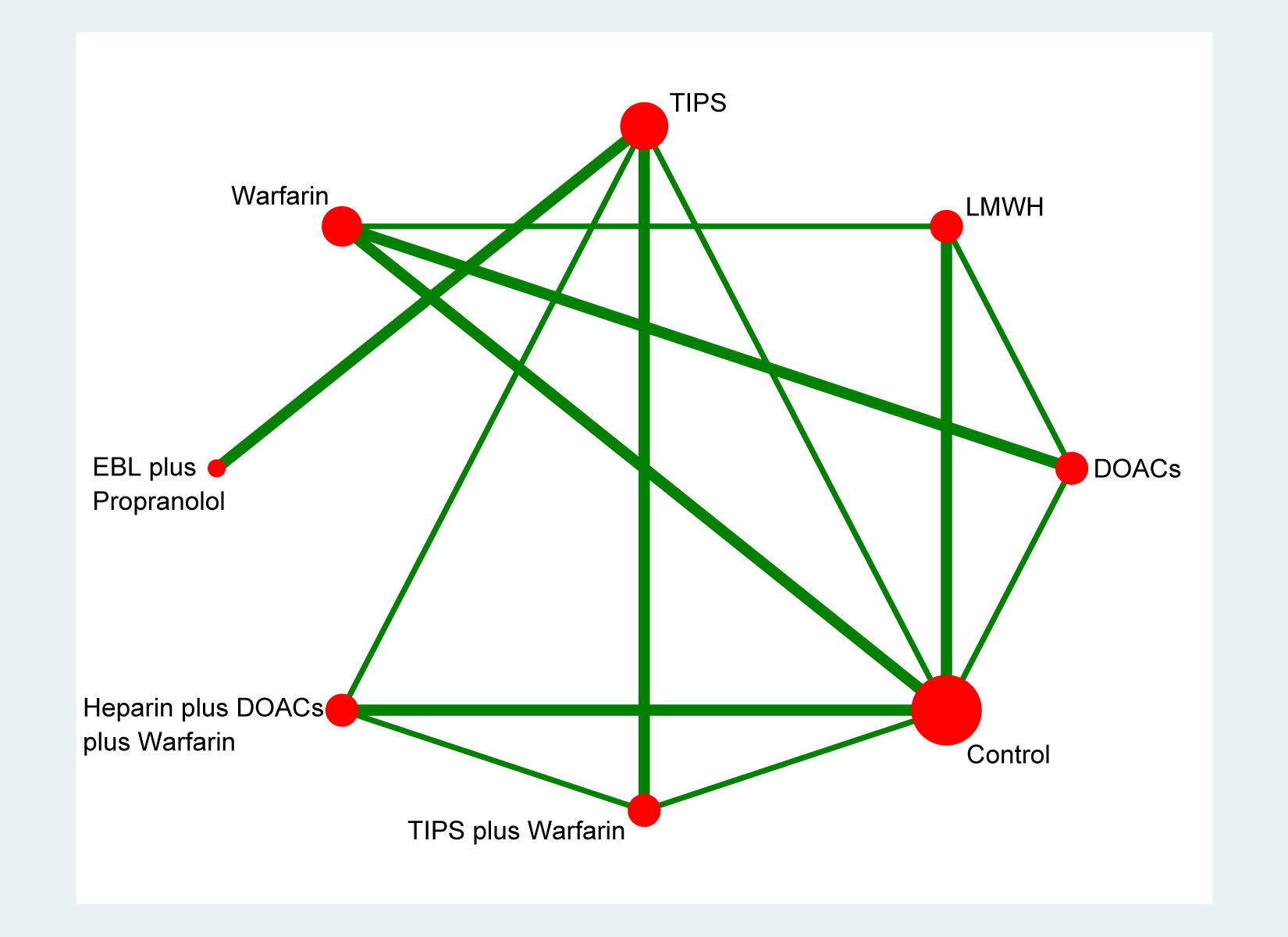


**Supplementary Figure 4** Network plot for PVT extension


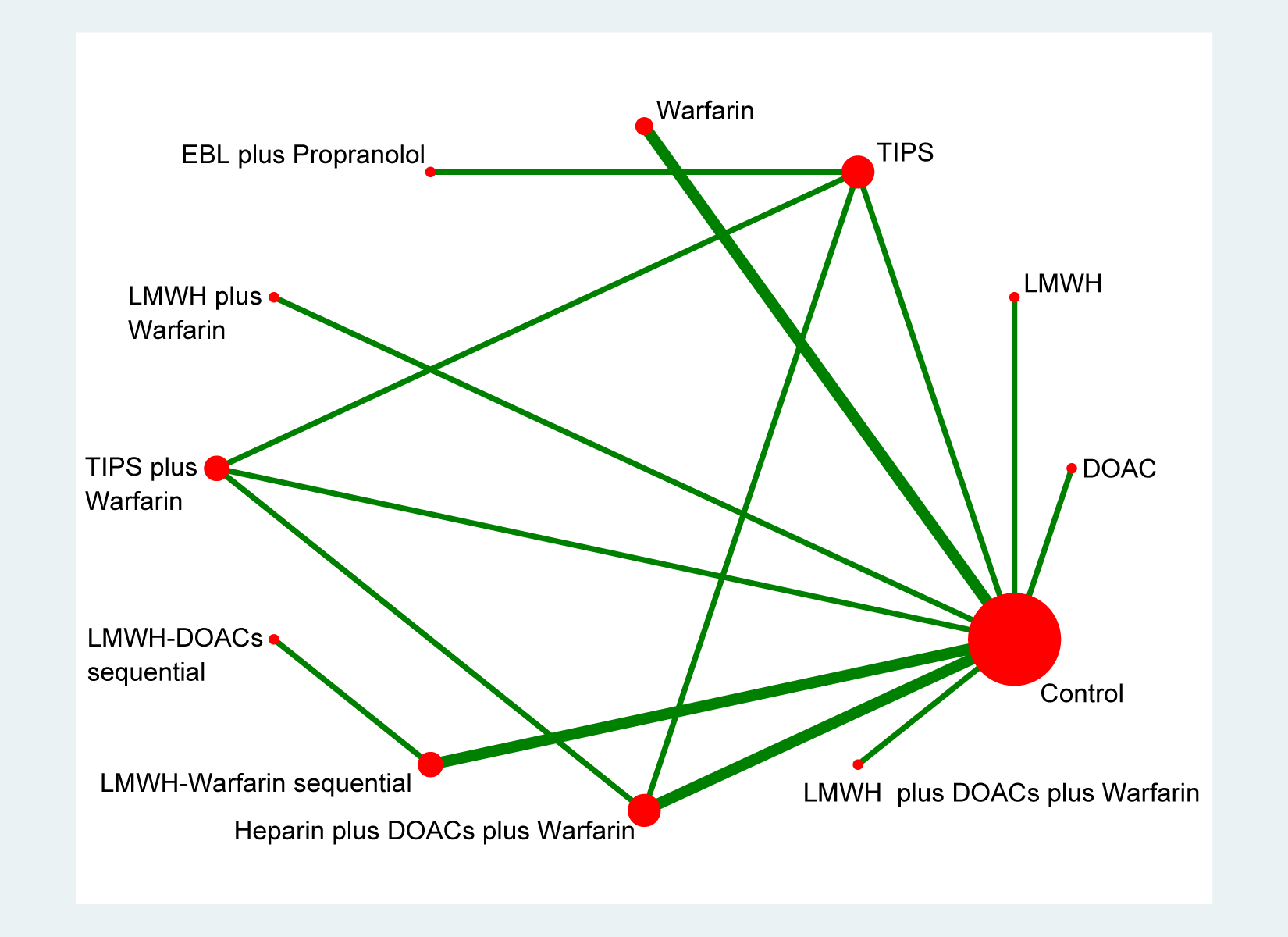


**Supplementary Figure 5** Network plot for hepatic encephalopathy


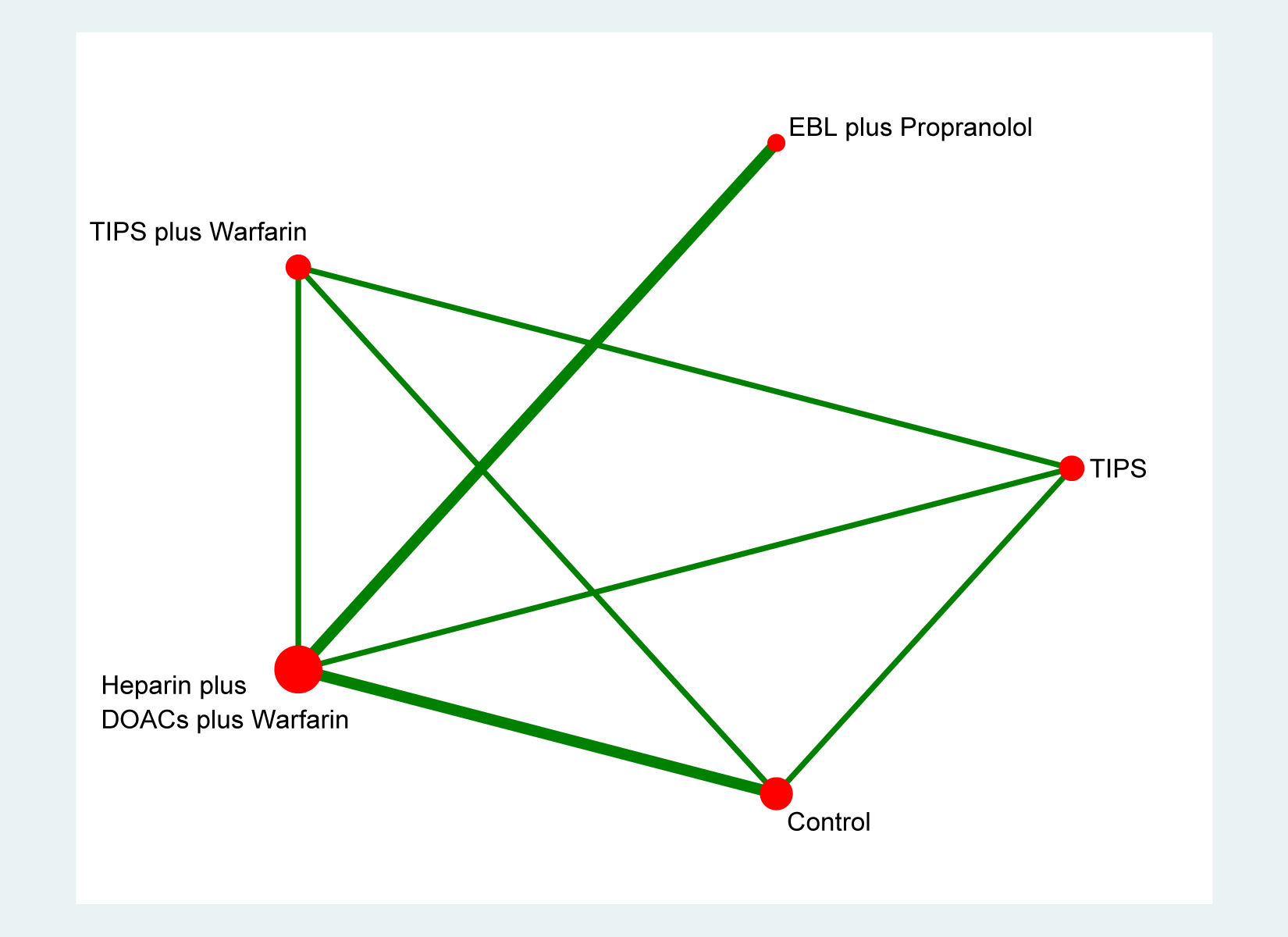


**Supplementary Figure 6** Funnel plot for complete recanalization

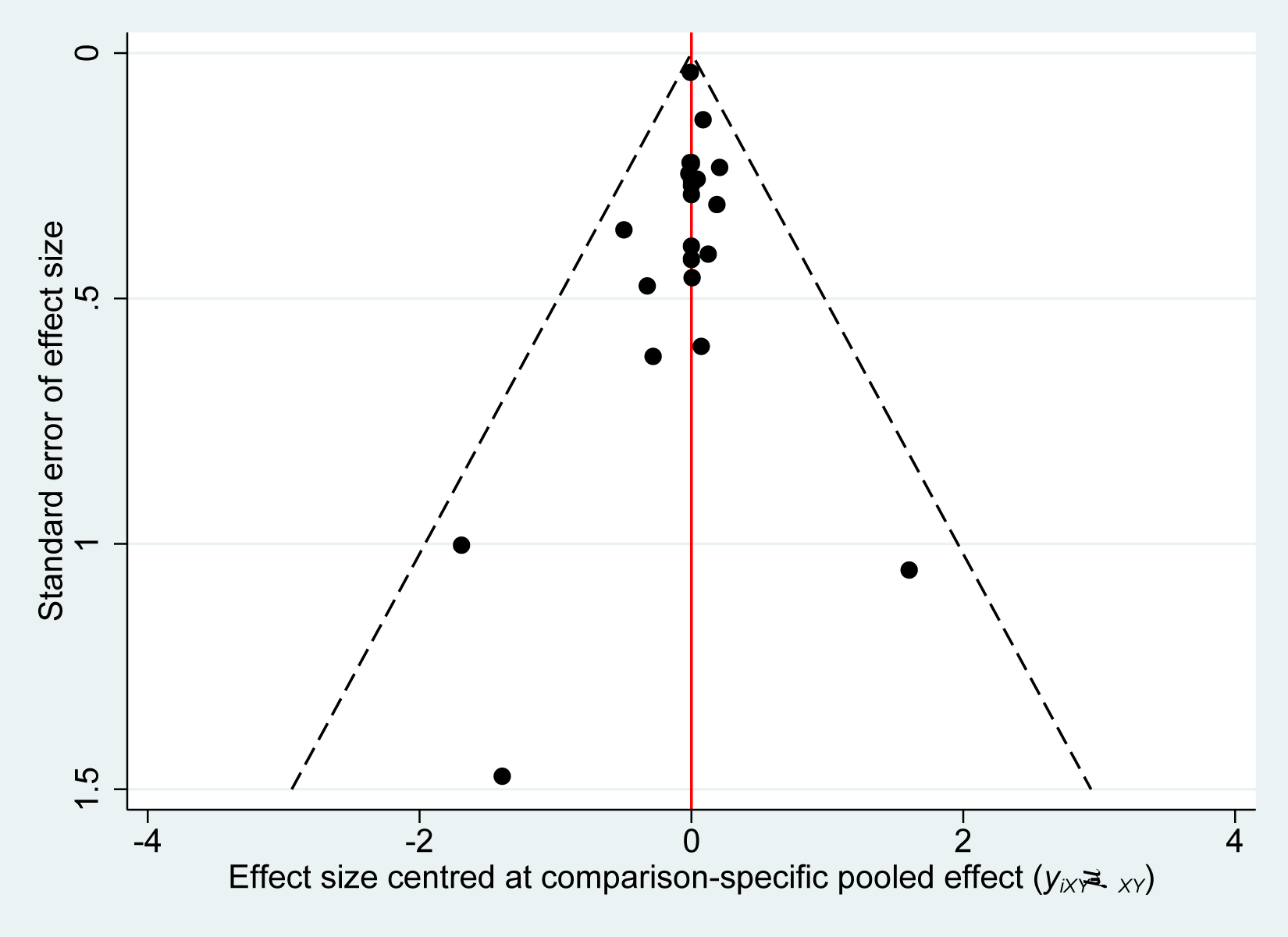


**Supplementary Figure 7** Funnel plot for partial recanalization

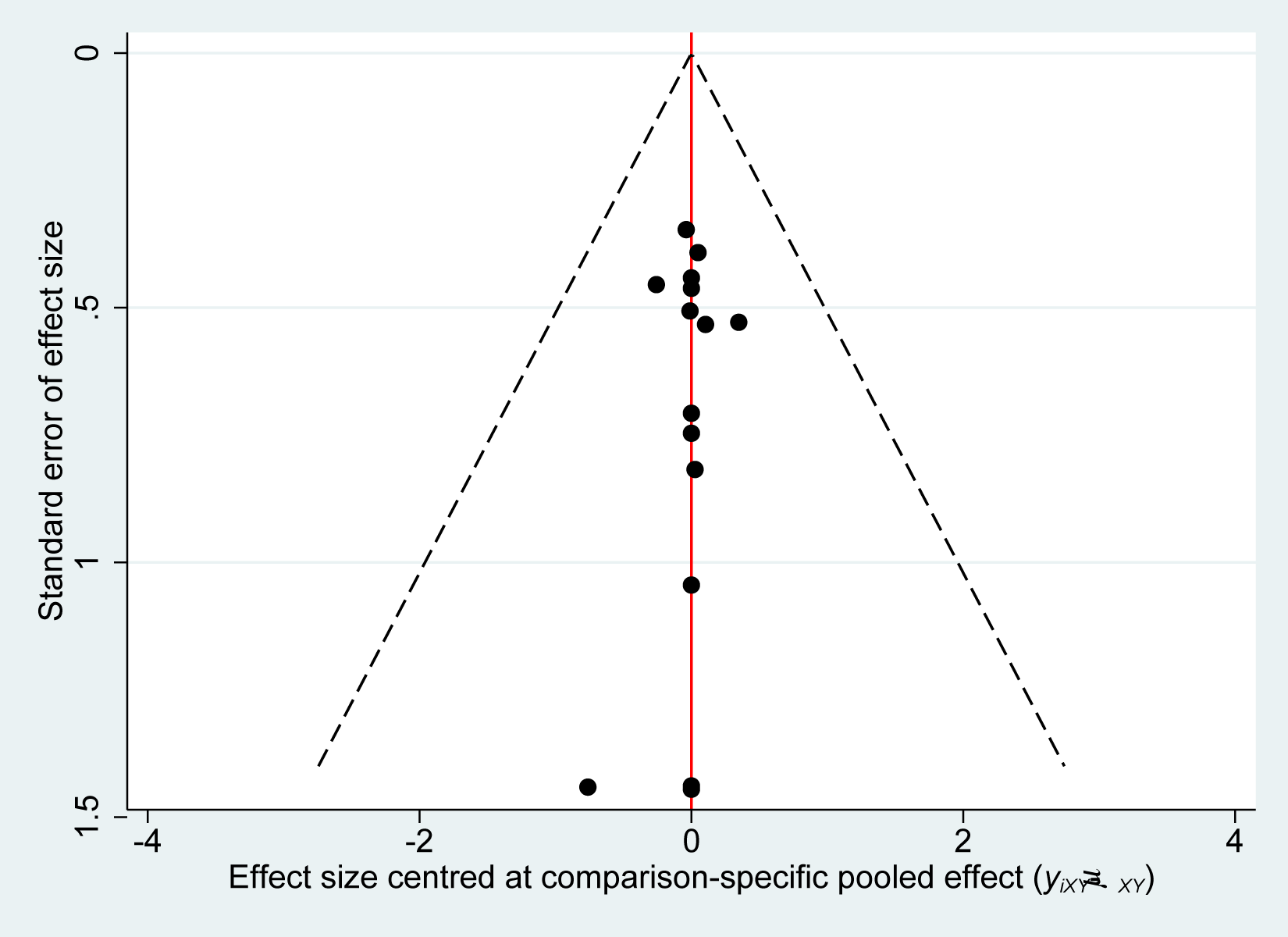


**Supplementary Figure 8** Funnel plot for bleeding

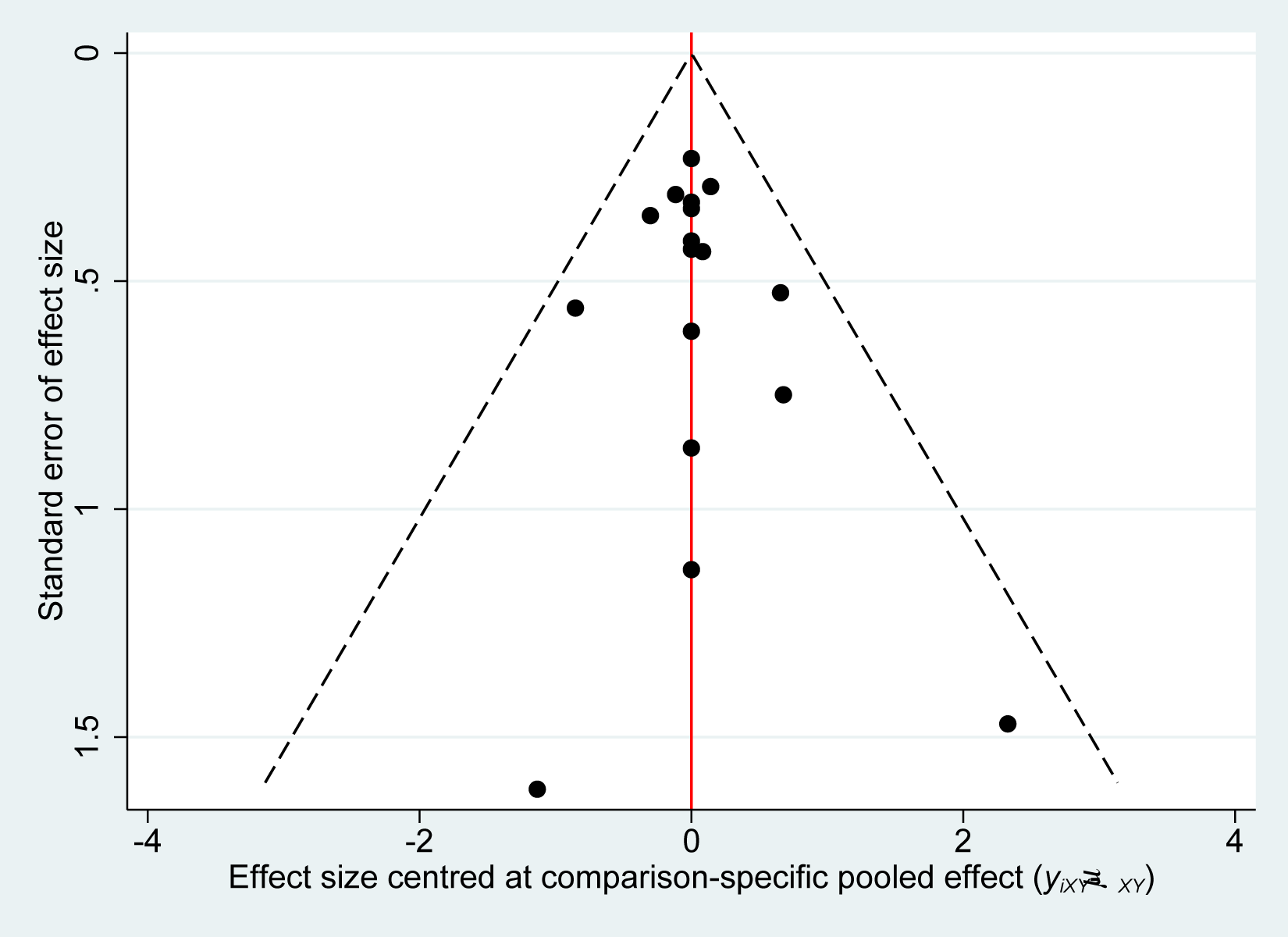


**Supplementary Figure 9** Funnel plot for major bleeding

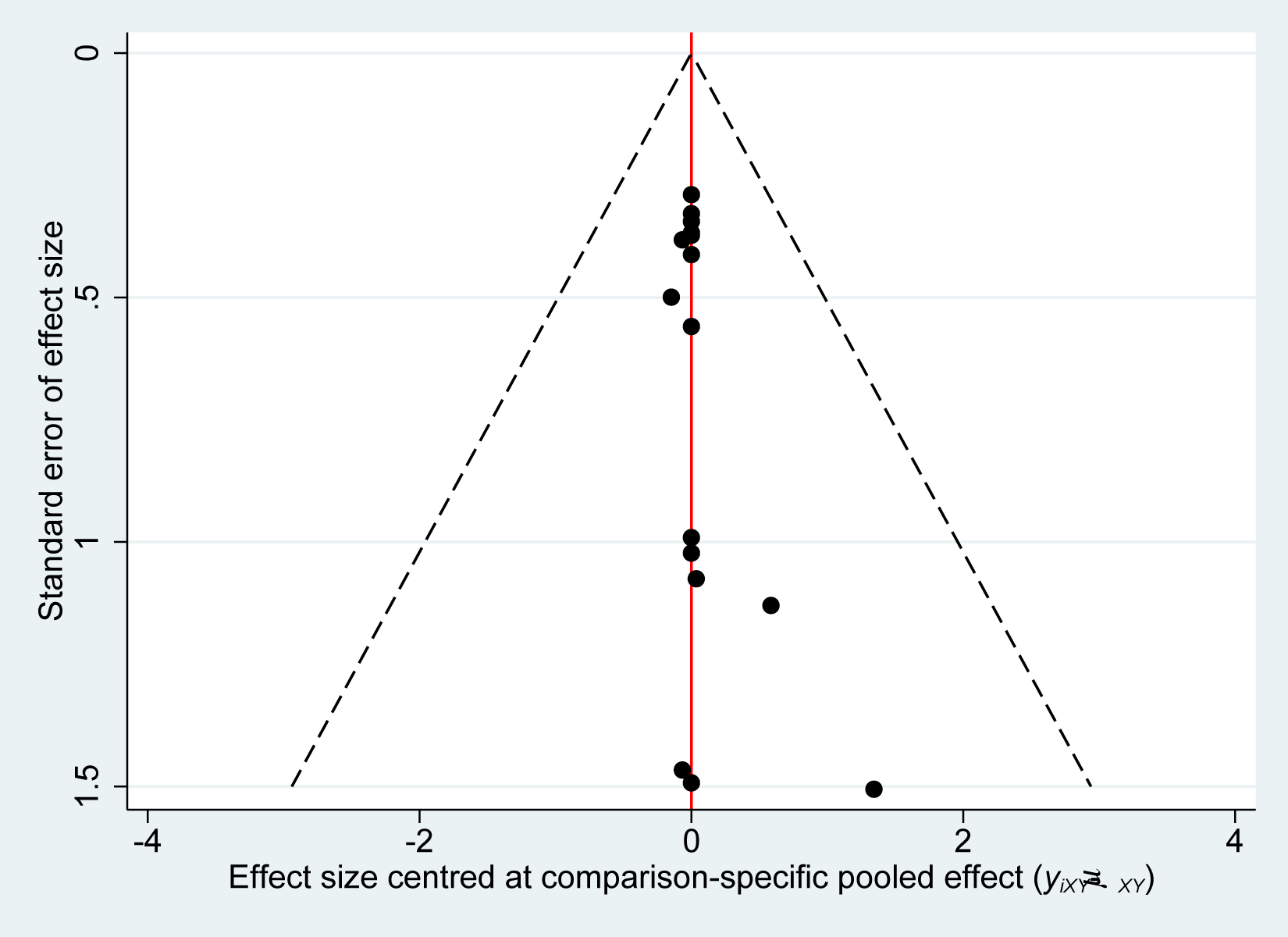


**Supplementary Figure 10** Funnel plot for mortality

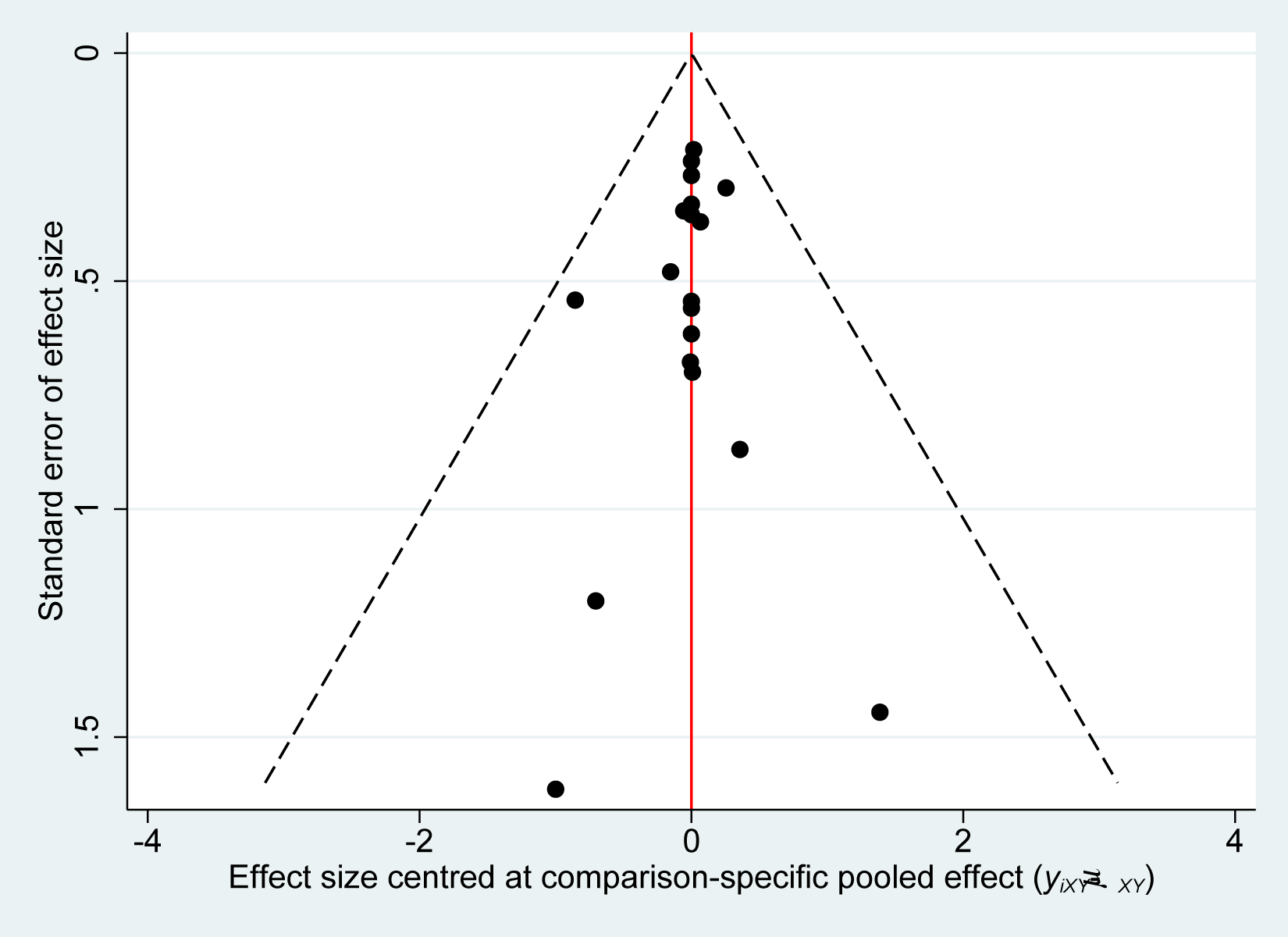


**Supplementary Figure 11** Funnel plot for PVT extension


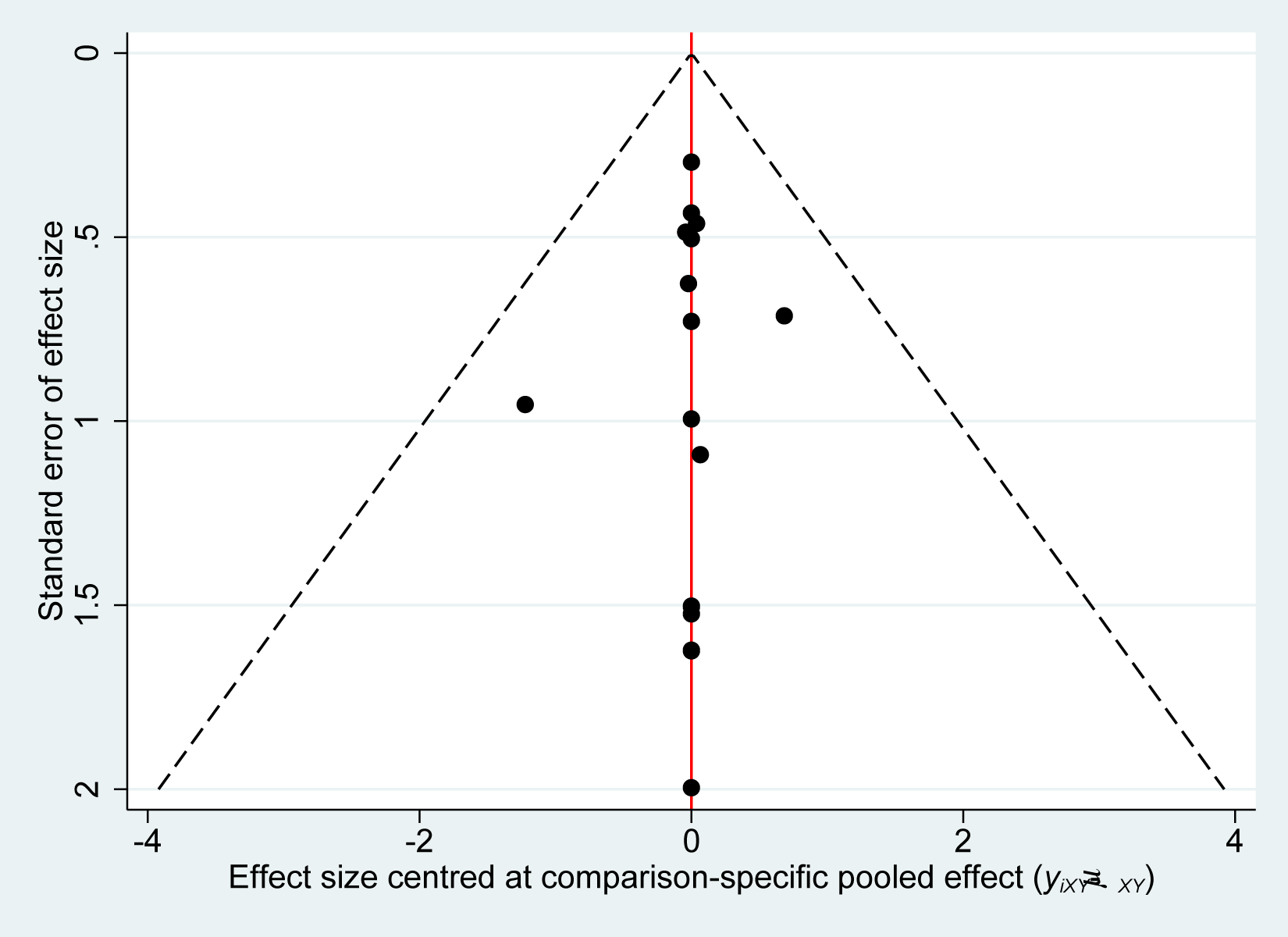


**Supplementary Figure 12** Funnel plot for hepatic encephalopathy


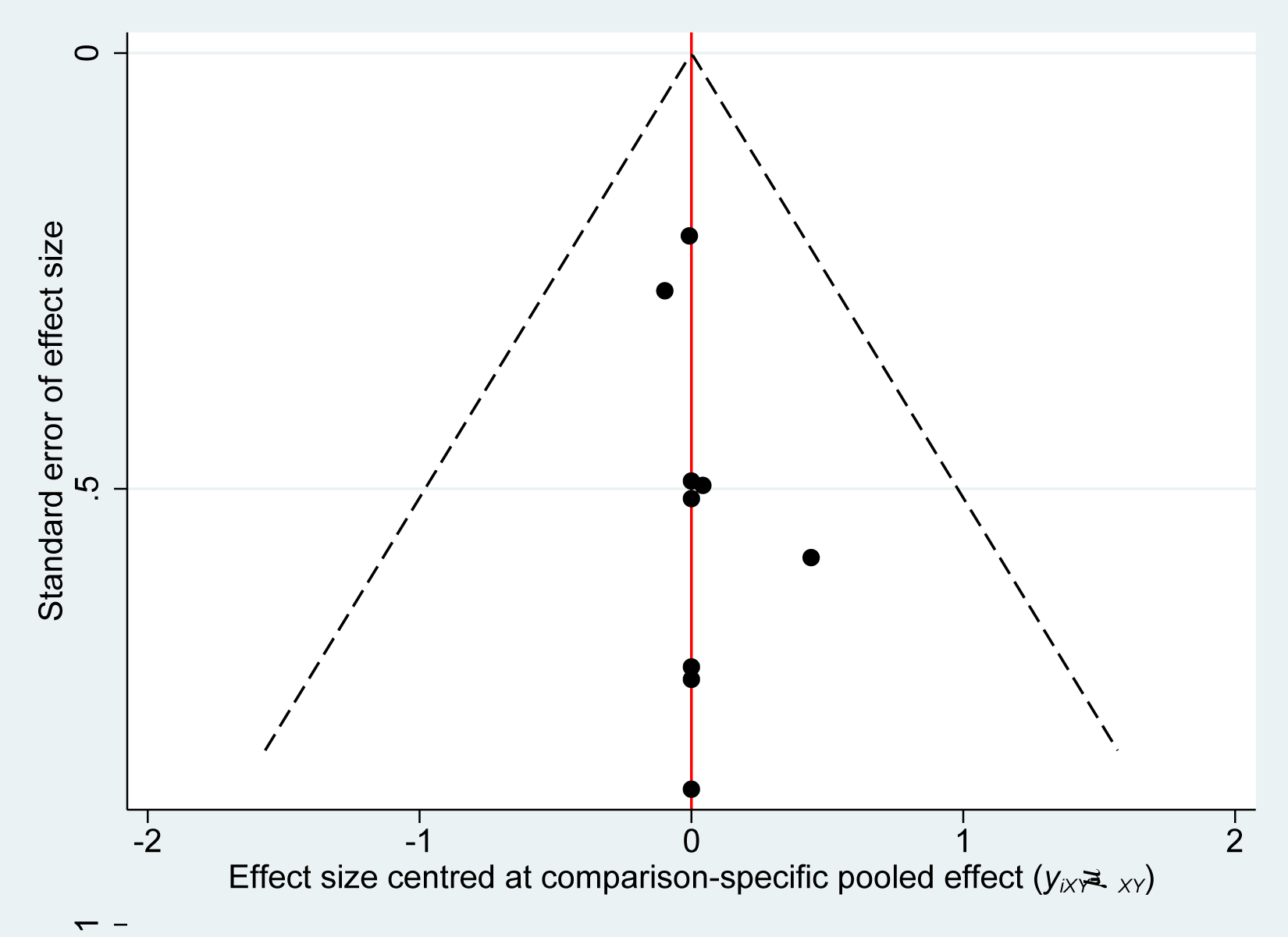

Supplement: Supplementary file 1 [file DataSheet1.docx]
